# Health promotion strategies for adolescents with suicidal behavior: a scoping review

**DOI:** 10.1590/1980-220X-REEUSP-2025-0338en

**Published:** 2026-09-07

**Authors:** Erika Augusta do Amaral Coelho Bezerra, Ivonete Teresinha Schulter Buss Heidemann, Aldalice Aguiar de Souza, Eliana Rosa da Fonseca, Camila Trindade Coelho, Paola Margarita Oñate Daza, Richard Augusto Thomann Beckert, Alaidistânia Aparecida Ferreira

**Affiliations:** 1 Universidade Federal de Santa Catarina, Florianópolis, SC, Brazil.; 2 Universidade do Estado do Amazonas, Manaus, AM, Brazil.; 3 Universidade Federal do Rio de Janeiro, Rio de Janeiro, RJ, Brazil.; 4 Universidade Federal do Amazonas, Manaus, AM, Brazil.

**Keywords:** Adolescent, Health Strategies, Suicide Prevention, Health Promotion, Suicide

## Abstract

**Objective:**

To map strategies for promoting the health of adolescents with suicidal behavior.

**Method:**

Scoping review in accordance with JBI methodological guidelines, without language or time limitations. Inclusion criteria for the studies: adolescents aged ten to 19 years with suicidal behavior to explore the concept of health promotion, in any health service, but without limiting this context. Sources consulted: MEDLINE/PubMed, PubMed Central/NLM, CINAHL, Academic Search Premier, Fonte Acadêmica/EBSCO, Scopus, Embase/Elsevier, LILACS, BDENF, INDEXPSI, CUMED, BINACIS, LIPECS, MedCarib, BBO, ARGMSAL, BDNPAR, BIGG, BRISA, HomeoIndex, PAHOIRIS, SES-SPBVS, SciELO, PsycInfo/APA.

**Results:**

Of the 22 articles selected, two strategic approaches emerged targeting adolescents: development of life skills – peer support, mental health literacy, youth engagement – and involvement of different social actors – family, professionals, community leaders, intersectoral support networks.

**Conclusion:**

Health promotion strategies were identified and mapped using two strategic approaches: first, they were directed at adolescents; then, they involved different social actors, broadening the scope of health promotion.

## INTRODUCTION

Health promotion strategies are gaining prominence in Primary Health Care (PHC) due to their strong convergence with its principles, which favor access to services and the implementation of integrated actions^(1)^. The PHC also allows the coordination of interventions aimed at both individual and collective issues, with the goal of improving living conditions and strengthening community health^(2,3)^. In this regard, health promotion (HP) broadens the concept of health and emerges as a promising strategy to address public health challenges by focusing on the determinants of health and enhancing intersectoral actions^(4,5)^.

With the aim of improving living and health conditions in both individual and collective dimensions, HP establishes a range of political strategies with cross-cutting guidelines and axes. Thus, the strategies are characterized by cooperation between different sectors and within the health sector itself, by coordination with social protection networks, and by encouraging broad participation and social control. Its main focus is to reduce vulnerabilities and risks resulting from multifaceted determinants – social, economic, political, cultural and environmental. Additionally, it prioritizes empowering individuals and the community to have greater control over their life process. Starting from a comprehensive understanding of the health-disease process and its determinants, HP seeks to integrate and value popular knowledge with technical and scientific knowledge through actions that encompass behavioral change, collective interventions, and the implementation of integrated public policies^(3,4)^.

In PHC, especially in the field of mental health, the need for care from the HP perspective, guided by the psychosocial paradigm, empowers people experiencing mental distress to become active subjects integrated into their communities, enabling new expressions of life, autonomy, and socialization(^6^).

People experiencing mental distress, especially children and adolescents, require broad, intersectoral strategies focused on developing social skills and competencies, strengthening, coping mechanisms, and social support, with a focus on HP^(4,7)^, considering their uniqueness and the territory in which they are located.

Among the various manifestations of psychological suffering, especially among children and adolescents, suicide stands out as one of its most serious forms, given its complexity and social impact, resulting from the neuropsychosocial and emotional immaturity of this population. From 2011 to 2022, there was evidence of an increase in suicide rates in all Brazilian regions, with a higher incidence among young people aged ten to 24, representing a growth of 6.14%^(8)^. In the Americas region, between 2000 and 2019, an increase of 17% in cases was observed, while in Brazil there was a significant 43% increase in the number of suicides^(9)^ during that same period.

Suicide among adolescents reflects multiple contributing factors, such as mental disorders, family conflicts, violence, bullying, social exclusion, substance use (alcohol and drugs), as well as the impact of technologies through media and social networks, which are frequently associated with negative effects on mental health^(10,11)^. Given this scenario, the need to identify strategies not only restricted to the prevention of harm, but also those that address the complexity of these public health problems, that are comprehensive and articulated with different sectors capable of facing these public health threats, becomes evident^(10,12)^.

Preliminary searches were conducted to find published reviews or ongoing protocols on the same topic, to ensure the originality of the study and avoid duplication of effort. This search included MEDLINE/PubMed, Cochrane Database of Systematic Reviews, Regional Portal of the Virtual Health Library (VHL), PROSPERO, and JBI Evidence Synthesis databases, identifying two review protocols: one focused on intersectoral care strategies for adolescents with suicidal behavior and self-harm, and another on interventions aimed at preventing and managing suicide and self-harm in educational settings. Although related to the topic, these studies do not address the specific focus of this proposal. Thus, no current or ongoing reviews were found that address health promotion strategies aimed at adolescents with suicidal behavior, which highlights a gap in knowledge and reinforces the relevance of this investigation. Therefore, this study aims to map health promotion strategies directed at adolescents with suicidal behavior.

## METHOD

### Design of Study

This is a scoping review conducted in accordance with JBI methodological guidelines^(13)^, aiming to ensure methodological rigor, clarity, and organization of the review process, and reported in accordance with guidelines of the PRISMA Extension for Scoping Reviews (PRISMA-ScR) checklist^(14)^. The protocol for this review was registered on the Open Science Framework (OSF) platform, with DOI https://doi.org/10.17605/OSF.IO/J9MQB.

### Research Question

The research question was defined in accordance with the acronym PCC (population/concept/context), where the population (P) is “adolescents with suicidal behavior”; the concept (C) alludes to “health promotion”; and the context (C) refers to any mental health services at all levels of care, but not limited to them. The following research question was established: What are the health promotion strategies for adolescents with suicidal behavior?

### Eligibility Criteria

Population – This review considers studies involving adolescents aged ten to 19 years^(15)^ with suicidal behavior. The World Health Organization (WHO)^(16)^ highlights suicide as a serious public health issue requiring a coordinated response. With timely, evidence-based, and low-cost interventions, it is possible to significantly reduce the occurrence of suicides. Although many incidents are impulsive, suicidal behavior is strongly associated with disasters, conflicts, violence, abuse or loss, and feelings of isolation. The WHO establishes effective, evidence-based interventions, which include the identification, assessment, management, and early follow-up of anyone affected by suicidal behavior^(16)^.

Concept – Studies addressing health promotion strategies for adolescents with suicidal behavior were included. The definition considered was that of the Ottawa Charter, which defines health promotion as “the process of enabling people to increase control over, and to improve, their health”^(4,17)^.

Context – The included studies were conducted in any location providing mental health services at any level of care. Services at any level of healthcare assistance, taking into account the age range of the participants, were considered equally, but not being limited to them.

Studies with experimental and quasi-experimental designs (randomized clinical trials, non-randomized controlled trials, before-and-after studies, and interrupted time series), analytical observational studies (prospective and retrospective cohorts, case-control studies, and analytical cross-sectional studies), and descriptive observational studies (case series, case reports, and descriptive cross-sectional studies) were considered. Qualitative studies (phenomenology, grounded theory, ethnography, qualitative description, and action research) were included, in addition to theoretical texts, opinion articles, dissertations, and theses. Systematic, scoping, and integrative reviews, as well as conference abstracts, letters, and editorials, were excluded.

### Search Strategy

The search strategy was carried out in three stages, aiming to locate published and unpublished studies, in collaboration with a specialized librarian certified by the JBI in review methods. Initially, a preliminary search was conducted in the following databases: MEDLINE/PubMed, Cochrane Database of Systematic Reviews, Regional Portal of the Virtual Health Library (VHL), PROSPERO and JBI Evidence Synthesis. In the second stage, the definitive strategy presented in Chart 1 was adapted for the following databases/portals: MEDLINE/PubMed, PubMed Central/NLM, CINAHL, Academic Search Premier, Fonte Acadêmica/EBSCO, Scopus, Embase/Elsevier, LILACS, BDENF, INDEXPSI, CUMED, BINACIS, LIPECS, MedCarib, BBO, ARGMSAL, BDNPAR, BIGG, BRISA, HomeoIndex, PAHOIRIS, SES-SPLILACS, BDENF, Index Psicologia/VHL, SciELO, and PsycInfo/APA. Grey literature was explored through the platform Science.gov; and the Google Scholar search tool was added. There were no restrictions on language or publication period. Finally, in the third stage, a manual search was conducted in the references of the included studies. The search was conducted on January 23, 2025.

**Chart 1:** Search strategies for databases/portals, Florianópolis – SC, Brazil, 2025.

Databases/portals	Strategies	Total
VHL	((adolescente OR adolescência OR adolescente* OR jovem OR jovens OR juventude OR joven OR jóvenes OR juventud OR jeunes OR jeunesse OR adolescent OR adolescence OR adolescent* OR teen* OR teenager* OR youth*) AND (“Ideação Suicida” OR “Ideación Suicida” OR “Ideas Suicidas” OR “Idéation suicidaire” OR “Idéation de suicide” OR “Idées suicidaires” OR “Comportamento Autodestrutivo” OR “Autoagressão Intencional” OR autolesão OR “Autolesão Intencional” OR “Autolesão não Suicida” OR “Conduta Autolesiva” OR “Ferimento Autoinfligido não Suicida” OR “Lesão Autoinfligida não Suicida” OR “Conducta Autodestructiva” OR “Autolesión Intencional” OR “Autolesión no Suicida” OR autolesiones OR “Herimiento Autoinferido no Suicida” OR “Herimientos Autoinferidos” OR “Comportement auto-agressif” OR “Automutilation délibérée” OR “Automutilation non suicidaire” OR “Comportement autoagressif” OR “Comportement autodestructeur” OR “Comportement d’auto-agression” OR “Comportement d’autoagression” OR “Comportement d’automutilation” OR suicídio OR suicídio* OR suicide OR “Suicidal Ideation” OR “Suicidal Ideations” OR “Self-Injurious Behavior” OR “Deliberate Self Harm” OR “Deliberate Self-Harm” OR “Intentional Self Harm” OR “Intentional Self Injuries” OR “Intentional Self Injury” OR “Non Suicidal Self Injury” OR “Non-Suicidal Self Injuries” OR “Non-Suicidal Self Injury” OR “Nonsuicidal Self Injuries” OR “Nonsuicidal Self Injury” OR “Self Destructive Behavior” OR “Self Harm” OR “Self Injurious Behavior” OR “Self Injury” OR “Self-Destructive Behavior” OR “Self-Destructive Behaviors” OR self-injuries OR “Self-Injurious Behaviors” OR self-injury OR suicide OR suicide* OR “Prevenção do Suicídio” OR “Conscientização sobre Suicídio” OR “Prevenção contra Suicídio” OR “Prevención del Suicidio” OR “Conciencia Sobre el Suicidio” OR “Prévention du suicide” OR “Suicide Prevention” OR “Suicide Awareness” OR “Suicide Preventions”)) AND ((“Promoção da Saúde” OR “Ambientes Apoiadores de Saúde” OR “Ambientes de Apoio à Saúde” OR “Campanhas de Saúde” OR “Item Promocional” OR “Itens Promocionais” OR “Programas de Bem-Estar” OR “Promoção do Bem Estar” OR “Promoção em Saúde” OR “Promoción de la Salud” OR “Ambientes de Apoyo a la Salud” OR “Campanas de Salud” OR “Entornos Apoyadores de la Salud” OR “Entornos de Apoyo a la Salud” OR “Items Promocionales” OR “Programas de Bienestar” OR “Promoción del Bienestar” OR “Promotion de la santé” OR “Campagnes de santé” OR “Campagnes pour la santé” OR “Points promotionnels” OR “Programmes de bien-être” OR “pessoas saudáveis” OR “programas para pessoas saudáveis” OR “Éducation Environnementale” OR “Educação em Saúde” OR “Educar para a Saúde” OR “Educação para a Saúde” OR “Educação Sanitária” OR “Educación en Salud” OR “Educación para la Salud” OR “Educación para la Salud Comunitaria” OR “Educación Sanitaria” OR “Éducation pour la santé” OR “Éducation communautaire en santé” OR “Éducation en santé” OR “Éducation pour la santé au sein de la communauté” OR “Éducation sanitaire” OR “Éducation sanitaire communautaire” OR “Formation communautaire en santé” OR “Health Promotion” OR “Health Campaign” OR “Health Campaigns” OR “Health Promotions” OR “Health-Supportive Environments” OR “Promotion of Health” OR “Promotional Item” OR “Promotional Items” OR “Wellness Program” OR “Wellness Programs” OR “healthy people 2010”or “healthy people programmes” OR “healthy people programs” OR “Health Education” OR “Community Health Education”)) AND db:(“LILACS” OR “BDENF” OR “INDEXPSI” OR “CUMED” OR “BINACIS” OR “LIPECS” OR “MedCarib” OR “BBO” OR “ARGMSAL” OR “BDNPAR” OR “BIGG” OR “BRISA” OR “HomeoIndex” OR “PAHOIRIS” OR “SES-SP”) AND instance:”lilacsplus”	714
Academic Searche Premier ( ) CINAHL with Full Text Fonte Acadêmica ( )	((TI Adolescent OR Adolescence OR Adolescent* OR Teen* OR Teenager* OR Youth* OR SU Adolescent OR Adolescence OR Adolescent* OR Teen* OR Teenager* OR Youth* OR AB Adolescent OR Adolescence OR Adolescent* OR Teen* OR Teenager* OR Youth*) AND (TI “Suicidal Ideation” OR “Suicidal Ideations” OR “Self-Injurious Behavior” OR “Deliberate Self Harm” OR “Deliberate Self-Harm” OR “Intentional Self Harm” OR “Intentional Self Injuries” OR “Intentional Self Injury” OR “Non Suicidal Self Injury” OR “Non-Suicidal Self Injuries” OR “Non-Suicidal Self Injury” OR “Nonsuicidal Self Injuries” OR “Nonsuicidal Self Injury” OR “Self Destructive Behavior” OR “Self Harm” OR “Self Injurious Behavior” OR “Self Injury” OR “Self-Destructive Behavior” OR “Self-Destructive Behaviors” OR Self-Injuries OR “Self-Injurious Behaviors” OR Self-Injury OR Suicide OR Suicide* OR “Suicide Prevention” OR “Awareness, Suicide” OR “Prevention, Suicide” OR “Suicide Awareness” OR “Suicide Preventions” OR SU “Suicidal Ideation” OR “Suicidal Ideations” OR “Self-Injurious Behavior” OR “Deliberate Self Harm” OR “Deliberate Self-Harm” OR “Intentional Self Harm” OR “Intentional Self Injuries” OR “Intentional Self Injury” OR “Non Suicidal Self Injury” OR “Non-Suicidal Self Injuries” OR “Non-Suicidal Self Injury” OR “Nonsuicidal Self Injuries” OR “Nonsuicidal Self Injury” OR “Self Destructive Behavior” OR “Self Harm” OR “Self Injurious Behavior” OR “Self Injury” OR “Self-Destructive Behavior” OR “Self-Destructive Behaviors” OR Self-Injuries OR “Self-Injurious Behaviors” OR Self-Injury OR Suicide OR Suicide* OR “Suicide Prevention” OR “Awareness, Suicide” OR “Prevention, Suicide” OR “Suicide Awareness” OR “Suicide Preventions” OR AB “Suicidal Ideation” OR “Suicidal Ideations” OR “Self-Injurious Behavior” OR “Deliberate Self Harm” OR “Deliberate Self-Harm” OR “Intentional Self Harm” OR “Intentional Self Injuries” OR “Intentional Self Injury” OR “Non Suicidal Self Injury” OR “Non-Suicidal Self Injuries” OR “Non-Suicidal Self Injury” OR “Nonsuicidal Self Injuries” OR “Nonsuicidal Self Injury” OR “Self Destructive Behavior” OR “Self Harm” OR “Self Injurious Behavior” OR “Self Injury” OR “Self-Destructive Behavior” OR “Self-Destructive Behaviors” OR Self-Injuries OR “Self-Injurious Behaviors” OR Self-Injury OR Suicide OR Suicide* OR “Suicide Prevention” OR “Awareness, Suicide” OR “Prevention, Suicide” OR “Suicide Awareness” OR “Suicide Preventions”)) AND (TI “Health Promotion” OR “Health Campaign” OR “Health Campaigns” OR “Health Promotions” OR “Health-Supportive Environments” OR “Promotion of Health” OR “Promotional Item” OR “Promotional Items” OR “Wellness Program” OR “Wellness Programs” OR “healthy people 2010”OR “healthy people programmes” OR “healthy people programs” OR “Health Education” OR “Community Health Education” OR SU “Health Promotion” OR “Health Campaign” OR “Health Campaigns” OR “Health Promotions” OR “Health-Supportive Environments” OR “Promotion of Health” OR “Promotional Item” OR “Promotional Items” OR “Wellness Program” OR “Wellness Programs” OR “healthy people 2010”OR “healthy people programmes” OR “healthy people programs” OR “Health Education” OR “Community Health Education” OR AB “Health Promotion” OR “Health Campaign” OR “Health Campaigns” OR “Health Promotions” OR “Health-Supportive Environments” OR “Promotion of Health” OR “Promotional Item” OR “Promotional Items” OR “Wellness Program” OR “Wellness Programs” OR “healthy people 2010”OR “healthy people programmes” OR “healthy people programs” OR “Health Education” OR “Community Health Education”)	1099
EMBASE	(‘adolescent’/exp OR adolescent OR ‘adolescence’/exp OR adolescence OR adolescent* OR teen* OR teenager* OR youth*) AND (‘suicidal ideation’/exp OR ‘suicidal ideation’ OR ‘suicidal ideations’ OR ‘self-injurious behavior’/exp OR ‘self-injurious behavior’ OR ‘deliberate self harm’/exp OR ‘deliberate self harm’ OR ‘deliberate self-harm’/exp OR ‘deliberate self-harm’ OR ‘intentional self harm’ OR ‘intentional self injuries’ OR ‘intentional self injury’ OR ‘non suicidal self injury’/exp OR ‘non suicidal self injury’ OR ‘non-suicidal self injuries’ OR ‘non-suicidal self injury’/exp OR ‘non-suicidal self injury’ OR ‘nonsuicidal self injuries’ OR ‘nonsuicidal self injury’/exp OR ‘nonsuicidal self injury’ OR ‘self destructive behavior’/exp OR ‘self destructive behavior’ OR ‘self harm’/exp OR ‘self harm’ OR ‘self injurious behavior’/exp OR ‘self injurious behavior’ OR ‘self-destructive behavior’ OR ‘self-destructive behaviors’ OR ‘self injuries’ OR ‘self-injurious behaviors’ OR ‘self injury’/exp OR ‘self injury’ OR ‘suicide’/exp OR suicide OR suicide* OR ‘suicide prevention’/exp OR ‘suicide prevention’ OR ‘awareness, suicide’ OR ‘prevention, suicide’ OR ‘suicide awareness’ OR ‘suicide preventions’) AND (‘health promotion’/exp OR ‘health promotion’ OR ‘health campaign’ OR ‘health campaigns’ OR ‘health promotions’ OR ‘health-supportive environments’ OR ‘promotion of health’ OR ‘promotional item’ OR ‘promotional items’/exp OR ‘promotional items’ OR ‘wellness program’ OR ‘wellness programs’ OR ‘healthy people 2010’/exp OR ‘healthy people 2010’ OR ‘healthy people programmes’/exp OR ‘healthy people programmes’ OR ‘healthy people programs’/exp OR ‘healthy people programs’ OR ‘health education’/exp OR ‘health education’ OR ‘community health education’) AND ([article]/lim OR [article in press]/lim OR [conference paper]/lim OR [conference review]/lim OR [review]/lim OR [preprint]/lim)	1825
Science	Title: (adolescent OR adolescence OR adolescent* OR teen* OR teenager* OR youth*) AND (“Suicidal Ideation” OR “Suicidal Ideations” OR “Self-Injurious Behavior” OR “Deliberate Self Harm” OR “Deliberate Self-Harm” OR “Intentional Self Harm” OR “Intentional Self Injuries” OR “Intentional Self Injury” OR “Non Suicidal Self Injury” OR “Non-Suicidal Self Injuries” OR “Non-Suicidal Self Injury” OR “Nonsuicidal Self Injuries” OR “Nonsuicidal Self Injury” OR “Self Destructive Behavior” OR “Self Harm” OR “Self Injurious Behavior” OR “Self Injury” OR “Self-Destructive Behavior” OR “Self-Destructive Behaviors” OR self-injuries OR “Self-Injurious Behaviors” OR self-injury OR suicide OR suicide* OR “Suicide Prevention” OR “Awareness, Suicide” OR “Prevention, Suicide” OR “Suicide Awareness” OR “Suicide Preventions”) AND (“Health Promotion” OR “Health Campaign” OR “Health Campaigns” OR “Health Promotions” OR “Health-Supportive Environments” OR “Promotion of Health” OR “Promotional Item” OR “Promotional Items” OR “Wellness Program” OR “Wellness Programs” OR “healthy people 2010” OR “healthy people programmes” OR “healthy people programs” OR “Health Education” OR “Community Health Education”)	270
PSYCINFO	(Title: adolescent OR Title: adolescence OR Title: adolescent* OR Title: teen* OR Title: teenager* OR Title: youth*) AND (Title: “Suicidal Ideation” OR Title: “Suicidal Ideations” OR Title: “Self-Injurious Behavior” OR Title: “Deliberate Self Harm” OR Title: “Deliberate Self-Harm” OR Title: “Intentional Self Harm” OR Title: “Intentional Self Injuries” OR Title: “Intentional Self Injury” OR Title: “Non Suicidal Self Injury” OR Title: “Non-Suicidal Self Injuries” OR Title: “Non-Suicidal Self Injury” OR Title: “Nonsuicidal Self Injuries” OR Title: “Nonsuicidal Self Injury” OR Title: “Self Destructive Behavior” OR Title: “Self Harm” OR Title: “Self Injurious Behavior” OR Title: “Self Injury” OR Title: “Self-Destructive Behavior” OR Title: “Self-Destructive Behaviors” OR Title: self-injuries OR Title: “Self-Injurious Behaviors” OR Title: self-injury OR Title: suicide OR Title: suicide* OR Title: “Suicide Prevention” OR Title: “Awareness, Suicide” OR Title: “Prevention, Suicide” OR Title: “Suicide Awareness” OR Title: “Suicide Preventions”) AND (Title: “Health Promotion” OR Title: “Health Campaign” OR Title: “Health Campaigns” OR Title: “Health Promotions” OR Title: “Health-Supportive Environments” OR Title: “Promotion of Health” OR Title: “Promotional Item” OR Title: “Promotional Items” OR Title: “Wellness Program” OR Title: “Wellness Programs” OR Title: “healthy people 2010” OR Title: “healthy people programmes” OR Title: “healthy people programs” OR Title: “Health Education” OR Title: “Community Health Education”) OR (Abstract: adolescent OR Abstract: adolescence OR Abstract: adolescent* OR Abstract: teen* OR Abstract: teenager* OR Abstract: youth*) AND (Abstract: “Suicidal Ideation” OR Abstract: “Suicidal Ideations” OR Abstract: “Self-Injurious Behavior” OR Abstract: “Deliberate Self Harm” OR Abstract: “Deliberate Self-Harm” OR Abstract: “Intentional Self Harm” OR Abstract: “Intentional Self Injuries” OR Abstract: “Intentional Self Injury” OR Abstract: “Non Suicidal Self Injury” OR Abstract: “Non-Suicidal Self Injuries” OR Abstract: “Non-Suicidal Self Injury” OR Abstract: “Nonsuicidal Self Injuries” OR Abstract: “Nonsuicidal Self Injury” OR Abstract: “Self Destructive Behavior” OR Abstract: “Self Harm” OR Abstract: “Self Injurious Behavior” OR Abstract: “Self Injury” OR Abstract: “Self-Destructive Behavior” OR Abstract: “Self-Destructive Behaviors” OR Abstract: self-injuries OR Abstract: “Self-Injurious Behaviors” OR Abstract: self-injury OR Abstract: suicide OR Abstract: suicide* OR Abstract: “Suicide Prevention” OR Abstract: “Awareness, Suicide” OR Abstract: “Prevention, Suicide” OR Abstract: “Suicide Awareness” OR Abstract: “Suicide Preventions”) AND (Abstract: “Health Promotion” OR Abstract: “Health Campaign” OR Abstract: “Health Campaigns” OR Abstract: “Health Promotions” OR Abstract: “Health-Supportive Environments” OR Abstract: “Promotion of Health” OR Abstract: “Promotional Item” OR Abstract: “Promotional Items” OR Abstract: “Wellness Program” OR Abstract: “Wellness Programs” OR Abstract: “healthy people 2010” OR Abstract: “healthy people programmes” OR Abstract: “healthy people programs” OR Abstract: “Health Education” OR Abstract: “Community Health Education”) OR (Keywords: adolescent OR Keywords: adolescence OR Keywords: adolescent* OR Keywords: teen* OR Keywords: teenager* OR Keywords: youth*) AND (Keywords: “Suicidal Ideation” OR Keywords: “Suicidal Ideations” OR Keywords: “Self-Injurious Behavior” OR Keywords: “Deliberate Self Harm” OR Keywords: “Deliberate Self-Harm” OR Keywords: “Intentional Self Harm” OR Keywords: “Intentional Self Injuries” OR Keywords: “Intentional Self Injury” OR Keywords: “Non Suicidal Self Injury” OR Keywords: “Non-Suicidal Self Injuries” OR Keywords: “Non-Suicidal Self Injury” OR Keywords: “Nonsuicidal Self Injuries” OR Keywords: “Nonsuicidal Self Injury” OR Keywords: “Self Destructive Behavior” OR Keywords: “Self Harm” OR Keywords: “Self Injurious Behavior” OR Keywords: “Self Injury” OR Keywords: “Self-Destructive Behavior” OR Keywords: “Self-Destructive Behaviors” OR Keywords: self-injuries OR Keywords: “Self-Injurious Behaviors” OR Keywords: self-injury OR Keywords: suicide OR Keywords: suicide* OR Keywords: “Suicide Prevention” OR Keywords: “Awareness, Suicide” OR Keywords: “Prevention, Suicide” OR Keywords: “Suicide Awareness” OR Keywords: “Suicide Preventions”) AND (Keywords: “Health Promotion” OR Keywords: “Health Campaign” OR Keywords: “Health Campaigns” OR Keywords: “Health Promotions” OR Keywords: “Health-Supportive Environments” OR Keywords: “Promotion of Health” OR Keywords: “Promotional Item” OR Keywords: “Promotional Items” OR Keywords: “Wellness Program” OR Keywords: “Wellness Programs” OR Keywords: “healthy people 2010” OR Keywords: “healthy people programmes” OR Keywords: “healthy people programs” OR Keywords: “Health Education” OR Keywords: “Community Health Education”)	205
PMC	(Title: (adolescent[Abstract] OR adolescence[Abstract] OR adolescent*[Abstract] OR teen*[Abstract] OR teenager*[Abstract] OR youth*)[Abstract] AND (“Suicidal Ideation”[Abstract] OR “Suicidal Ideations”[Abstract] OR “Self-Injurious Behavior”[Abstract] OR “Deliberate Self Harm”[Abstract] OR “Deliberate Self-Harm”[Abstract] OR “Intentional Self Harm”[Abstract] OR “Intentional Self Injuries”[Abstract] OR “Intentional Self Injury”[Abstract] OR “Non Suicidal Self Injury”[Abstract] OR “Non-Suicidal Self Injuries”[Abstract] OR “Non-Suicidal Self Injury”[Abstract] OR “Nonsuicidal Self Injuries”[Abstract] OR “Nonsuicidal Self Injury”[Abstract] OR “Self Destructive Behavior”[Abstract] OR “Self Harm”[Abstract] OR “Self Injurious Behavior”[Abstract] OR “Self Injury”[Abstract] OR “Self-Destructive Behavior”[Abstract] OR “Self-Destructive Behaviors”[Abstract] OR self-injuries[Abstract] OR “Self-Injurious Behaviors”[Abstract] OR self-injury[Abstract] OR suicide[Abstract] OR suicide*[Abstract] OR “Suicide Prevention”[Abstract] OR “Awareness, Suicide”[Abstract] OR “Prevention, Suicide”[Abstract] OR “Suicide Awareness”[Abstract] OR “Suicide Preventions”)[Abstract] AND (“Health Promotion”[Abstract] OR “Health Campaign”[Abstract] OR “Health Campaigns”[Abstract] OR “Health Promotions”[Abstract] OR “Health-Supportive Environments”[Abstract] OR “Promotion of Health”[Abstract] OR “Promotional Item”[Abstract] OR “Promotional Items”[Abstract] OR “Wellness Program”[Abstract] OR “Wellness Programs”[Abstract] OR “healthy people 2010”[Abstract] OR “healthy people programmes”[Abstract] OR “healthy people programs”[Abstract] OR “Health Education”[Abstract] OR “Community Health Education”)[Abstract])	10
PUBMED	(Adolescent[mh] OR Adolescence OR Adolescent* OR Teen* OR Teenager* OR Youth*) AND (“Suicidal Ideation”[mh] OR “Suicidal Ideations” OR “Self-Injurious Behavior”[mh] OR “Deliberate Self Harm” OR “Deliberate Self-Harm” OR “Intentional Self Harm” OR “Intentional Self Injuries” OR “Intentional Self Injury” OR “Non Suicidal Self Injury” OR “Non-Suicidal Self Injuries” OR “Non-Suicidal Self Injury” OR “Nonsuicidal Self Injuries” OR “Nonsuicidal Self Injury” OR “Self Destructive Behavior” OR “Self Harm” OR “Self Injurious Behavior” OR “Self Injury” OR “Self-Destructive Behavior” OR “Self-Destructive Behaviors” OR Self-Injuries OR “Self-Injurious Behaviors” OR Self-Injury OR Suicide[mh] OR Suicide* OR “Suicide Prevention”[mh] OR “Suicide Awareness” OR “Suicide Preventions”) AND (“Health Promotion”[mh] OR Health Campaign*[tiab] OR Health Promotion*[tiab] OR “Health-Supportive Environments”[tiab] OR “Promotion of Health”[tiab] OR Promotional Item*[tiab] OR Wellness Program*[tiab] OR “healthy people”[tiab] OR healthy people program*[tiab] OR “Health Education”[mh] OR Health Education[tiab])	983
SCIELO	(adolescent OR adolescence OR adolescent* OR teen* OR teenager* OR youth*) AND (“Suicidal Ideation” OR “Suicidal Ideations” OR “Self-Injurious Behavior” OR “Deliberate Self Harm” OR “Deliberate Self-Harm” OR “Intentional Self Harm” OR “Intentional Self Injuries” OR “Intentional Self Injury” OR “Non Suicidal Self Injury” OR “Non-Suicidal Self Injuries” OR “Non-Suicidal Self Injury” OR “Nonsuicidal Self Injuries” OR “Nonsuicidal Self Injury” OR “Self Destructive Behavior” OR “Self Harm” OR “Self Injurious Behavior” OR “Self Injury” OR “Self-Destructive Behavior” OR “Self-Destructive Behaviors” OR self-injuries OR “Self-Injurious Behaviors” OR self-injury OR suicide OR suicide* OR “Suicide Prevention” OR “Awareness, Suicide” OR “Prevention, Suicide” OR “Suicide Awareness” OR “Suicide Preventions”) AND (“Health Promotion” OR “Health Campaign” OR “Health Campaigns” OR “Health Promotions” OR “Health-Supportive Environments” OR “Promotion of Health” OR “Promotional Item” OR “Promotional Items” OR “Wellness Program” OR “Wellness Programs” OR “healthy people 2010” OR “healthy people programmes” OR “healthy people programs” OR “Health Education” OR “Community Health Education”)	6
SCOPUS	(TITLE-ABS-KEY (adolescent OR adolescence OR adolescent* OR teen* OR teenager* OR youth*) AND TITLE-ABS-KEY (“Suicidal Ideation” OR “Suicidal Ideations” OR “Self-Injurious Behavior” OR “Deliberate Self Harm” OR “Deliberate Self-Harm” OR “Intentional Self Harm” OR “Intentional Self Injuries” OR “Intentional Self Injury” OR “Non Suicidal Self Injury” OR “Non-Suicidal Self Injuries” OR “Non-Suicidal Self Injury” OR “Nonsuicidal Self Injuries” OR “Nonsuicidal Self Injury” OR “Self Destructive Behavior” OR “Self Harm” OR “Self Injurious Behavior” OR “Self Injury” OR “Self-Destructive Behavior” OR “Self-Destructive Behaviors” OR self-injuries OR “Self-Injurious Behaviors” OR self-injury OR suicide OR suicide* OR “Suicide Prevention” OR “Awareness, Suicide” OR “Prevention, Suicide” OR “Suicide Awareness” OR “Suicide Preventions”) AND TITLE-ABS-KEY (“Health Promotion” OR “Health Campaign” OR “Health Campaigns” OR “Health Promotions” OR “Health-Supportive Environments” OR “Promotion of Health” OR “Promotional Item” OR “Promotional Items” OR “Wellness Program” OR “Wellness Programs” OR “healthy people 2010” OR “healthy people programmes” OR “healthy people programs” OR “Health Education” OR “Community Health Education”))	1133
WOS	TS=(Adolescent OR Adolescence OR Adolescent* OR Teen* OR Teenager* OR Youth*) AND TS=(“Suicidal Ideation” OR “Suicidal Ideations” OR “Self-Injurious Behavior” OR “Deliberate Self Harm” OR “Deliberate Self-Harm” OR “Intentional Self Harm” OR “Intentional Self Injuries” OR “Intentional Self Injury” OR “Non Suicidal Self Injury” OR “Non-Suicidal Self Injuries” OR “Non-Suicidal Self Injury” OR “Nonsuicidal Self Injuries” OR “Nonsuicidal Self Injury” OR “Self Destructive Behavior” OR “Self Harm” OR “Self Injurious Behavior” OR “Self Injury” OR “Self-Destructive Behavior” OR “Self-Destructive Behaviors” OR Self-Injuries OR “Self-Injurious Behaviors” OR Self-Injury OR Suicide OR Suicide* OR “Suicide Prevention” OR “Awareness, Suicide” OR “Prevention, Suicide” OR “Suicide Awareness” OR “Suicide Preventions”) AND TS=(“Health Promotion” OR “Health Campaign” OR “Health Campaigns” OR “Health Promotions” OR “Health-Supportive Environments” OR “Promotion of Health” OR “Promotional Item” OR “Promotional Items” OR “Wellness Program” OR “Wellness Programs” OR “healthy people 2010” OR “healthy people programmes” OR “healthy people programs” OR “Health Education” OR “Community Health Education”)	338
TOTAL		6583

### Selection of Studies

After searching the databases, the identified references were uploaded to EndNote 21 (Clarivate Analytics, PA, USA), and duplicates were removed. Next, these references were exported to the software Intelligent Systematic Review (Rayyan) for managing the selection process. The documents were evaluated by title and abstract based on the inclusion criteria, a process conducted by four independent reviewers, following a pilot test. Following evaluation, the relevant sources were retrieved and fully assessed, and the decisions were recorded in Rayyan.

The disagreements arising among the reviewers at each stage of the selection process were resolved through discussion, consensus, and even a fifth reviewer. The results of the search and study inclusion process are presented in the flowchart, in accordance with the PRISMA-ScR guidelines^(14)^.

### Data Extraction and Analysis

Data were extracted from the included articles by four independent reviewers using a data extraction tool (Chart 2) developed by the reviewers, based on the JBI manual. Data included specific details about the characteristics of the studies (citation, year, country, objective, type of study, sample), participants, concept, context (PCC), study methods, and main conclusions relevant to the review question.

**Chart 2:** Characteristics of the studies included in the scoping review – Florianópolis, SC, Brazil, 2025.

Citation/year/ country		Objective	Study method/type	Sample/participants	Intervention scenario/context	Main results/strategies
1	Klingman and Hochdorf^(18)^, 1993, Israel.	To evaluate the effectiveness of a school-based primary prevention program and the empowerment of students as guardians against self-destructive behaviors.	Quasi-experimental study with an intervention group and a control group.	237 students in the 8th grade (12 to 13 years old)	Public school	Skills for life Peer support
2	Eggert et al.^(19)^, 1994, United States.	To evaluate the effectiveness of a school-based primary prevention program aimed at reducing the risk of suicide and drug use.	Quasi-experimental study with an intervention group and a control group.	Approximately 1,000 students from five high schools	Public school	Skills for life Extracurricular activities Peer support
3	LaFromboise and Howard-Pitney^(20)^, 1995, United States.	To evaluate the effectiveness of a suicide prevention program focused on life skills.	Quasi-experimental study with an intervention group and a control group.	128 high school students (ages 14 to 19)	Public school	Skills for life
4	Coggan et al.^(21)^, 1997, New Zealand.	To investigate young people’s opinions on suicide among young people.	Qualitative study (focus groups)	140 young people between the ages of 16 and 24	Community, schools, universities	Skills for life Training for teachers and other professionals Educational campaign Engagement and protagonism Social support and support network
5	Waring et al.^(22)^, 2000, Australia.	To describe several projects of the Hunter Institute of Mental Health, focusing on the promotion and prevention of mental health in adolescents.	Experience report / Descriptive.	There is no specific sample of participants, but it includes descriptions of projects aimed at high school and university students.	School environments (high schools and universities), health services, and the general community.	Mental health literacy Social support and support network Training for teachers and other professionals Culture and art Active/multimodal methodology
6	Day^(23)^, 2004, United Kingdom.	To investigate whether teaching problem-solving skills to adolescents can be an effective strategy in preventing youth suicide.	Action research with a qualitative and quantitative approach.	Students aged 11 to 12 in the 7th grade, along with teachers, school nurses, and mental health professionals.	School environment	Skills for life Intersectoral coordination
7	Armstrong^(24)^, 2011, Canada.	To analyze the risk and protective factors associated with suicidal ideation in adolescents from rural and urban contexts and to propose intervention strategies.	Quantitative, cross-sectional, descriptive, and analytical study.	813 high school students: 459 of rural areas and 354 of urban areas	School and community environment in rural and urban areas.	Engagement and protagonism Extracurricular activities
8	Carli^(25)^, 2016, Switzerland.	To develop and evaluate the effectiveness of an online intervention promoting mental health and preventing suicide, aimed at European teenagers, through the project SUPREME.	Randomized, multicenter, single-blind clinical trial.	2,286 students, aged 14 to 18, from six European countries: Sweden, Italy, England, Spain, Lithuania, Estonia, and Hungary.	School environment and online (virtual platform developed specifically for the project SUPREME)	Digital platform
9	Silverstone et al.^(26)^, 2017, Canada.	To evaluate the long-term outcomes of a multimodal school program to reduce suicidal ideation, depression, and anxiety among adolescents.	Quantitative, longitudinal, quasi-experimental study.	Initially, 6,227 students Age range: 11 to 18 years (6th to 12th grade). 1,884 completed all four assessments.	Public schools	Skills for life Active/multimodal methodology Intersectoral coordination
10	Kahn et al.^(27)^, 2020, Germany.	To understand how the Youth Aware of Mental Health program, a universal school-based suicide prevention intervention, interacts with different coping strategies.	Prospective randomized clinical trial	5,654 students, aged between 14 and 16, from 85 schools in ten European countries.	Schools in Europe	Extracurricular activities Socio-emotional skills and competence
11	Schilling et al.^(28)^, 2021, Chile.	To determine how adolescents’ life experiences contributed to their performance as experts by experience and to the development of the Clan Project, aimed at promoting mental health and preventing suicide.	Qualitative case study	Six students, aged between 13 and 20.	Schools and digital environments in Chile	Peer support Digital platform Leadership development
12	McGillivray et al.^(29)^, 2021, Australia.	To evaluate the impact of the universal Youth Aware of Mental Health program on promoting mental health and preventing suicide.	Single-arm study with pre- and post-testing, a quasi-experimental study	556 ninth-grade students (aged 13-16) from 18 schools in four regions of New South Wales, Australia.	High schools.	Skills for life Active/multimodal methodology
13	Kaur^(30)^ 2021, India	To analyze the effect of life skills training on adolescents’ suicidal behavior.	Quasi-experimental study (with a control group and an intervention group - pre-and posttest)	970 students from the 9th grade of high school, from 11 schools	Public high schools	Skills for life Support network
14	Chou et al.^(31)^, 2022, Canada.	To evaluate the effectiveness of the program Core Connectors Initiative.	Mixed methods study	29 students, aged between 15 and 17	A public school, a private school, and a community center.	Mental health literacy Peer support Support network
15	Chartier et al.^(32)^, 2022, Canada.	To analyze, from the perspective of young people, mentors, and elders from indigenous communities, the PAX Dream Makers approach, aimed at promoting mental health and preventing suicide among young people.	Qualitative study, of the case study type.	30 teenagers (12 to 18 years old) 17 adult mentors and elders from six remote indigenous communities	Indigenous First Nations communities in Manitoba, in the school and community context.	Skills for life Digital platform Leadership development
16	Libon et al.^(33)^, 2023, Canada.	To gather and document young people’s perspectives on the design and development of a peer support model for promoting mental health and preventing suicide.	Descriptive qualitative study, with an approach of participatory codesign, based on the socio-ecological structure	11 young people, aged between 15 and 24 years old	Rural communities in western Canada	Peer support Digital platform
17	Walsh et al.^(34)^, 2023, Ireland.	To explore young people’s perspectives on promoting school mental health and preventing suicide, using a participatory approach (Photovoice).	A qualitative, participatory study with reflective and experiential thematic analysis, grounded in a critical approach.	15 teenagers aged 16 to 19	School and community environment in Ireland	Peer support
18	Biber and Brandenburg^(35)^, 2024, United States.	To describe the implementation process of the Sources of Strength program in a high school, aiming to strengthen protective factors for mental health and prevent suicidal behavior and opioid use.	Case study with process evaluation.	1,250 high school students. 135 high school students acted as peer leaders (10.8% of the school). 15 university mentors, 13 teachers and high school staff as adult mentors.	High school	Intersectoral coordination Training for teachers and other professionals
19	Bohr et al.^(36)^, 2024. Canada.	To culturally adapt and evaluate the SPARX digital program for Inuit youth, aiming to strengthen mental health, resilience, and reduce suicide risk.	Participatory action research, using mixed methods.	121 young people, aged 13 to 24. 27 youth leaders (aged 15 to 26)	School environment, community environment, including virtual spaces.	Skills for life Culture and art Digital platform
20	Knappe et al.^(37)^, 2024, Germany.	To evaluate the effectiveness of the HEYLiFE school suicide prevention program.	Quasi-experimental, comparative, within-group study (pre- and post-intervention)	218 students, aged 14 to 18	Public schools	Mental health literacy Active/multimodal methodology Skills for life
21	Perrino et al.^(38)^, 2024, United States.	To adapt a digital intervention to promote the mental health of Hispanic adolescents, focusing on reducing symptoms of depression, anxiety, and suicide risk.	Study of intervention development and adaptation.	16 teenagers (aged 12 to 17) and 16 parents.	Primary care clinics.	Skills for life Digital platform Family involvement
22	Wexler et al.^(39)^, 2025, United States.	To analyze how self-efficacy in well-being, self-efficacy in suicide prevention, and participation in communities of practice influence suicide prevention behaviors and the promotion of well-being.	Cross-sectional, quantitative study	398 participants (aged 15 to 29)	Rural and remote indigenous communities, in community spaces and local social networks.	Intersectoral coordination Involvement of community and family leaders

Data that answered the objective of the review were analyzed and presented through a narrative summary, which described the results related to the research question. It is important to emphasize that they were presented in table form based on the categorized data to facilitate understanding.

### Ethical Aspects

This scoping review followed applicable methodological and ethical guidelines, respecting best practices in scientific research and the copyright of the included studies. Since this analysis involved publicly available secondary data without the involvement of human beings or identifiable information, there was no need to submit it to the Research Ethics Committee or obtain Free Informed Consent.

## RESULTS

**Figure 1:**
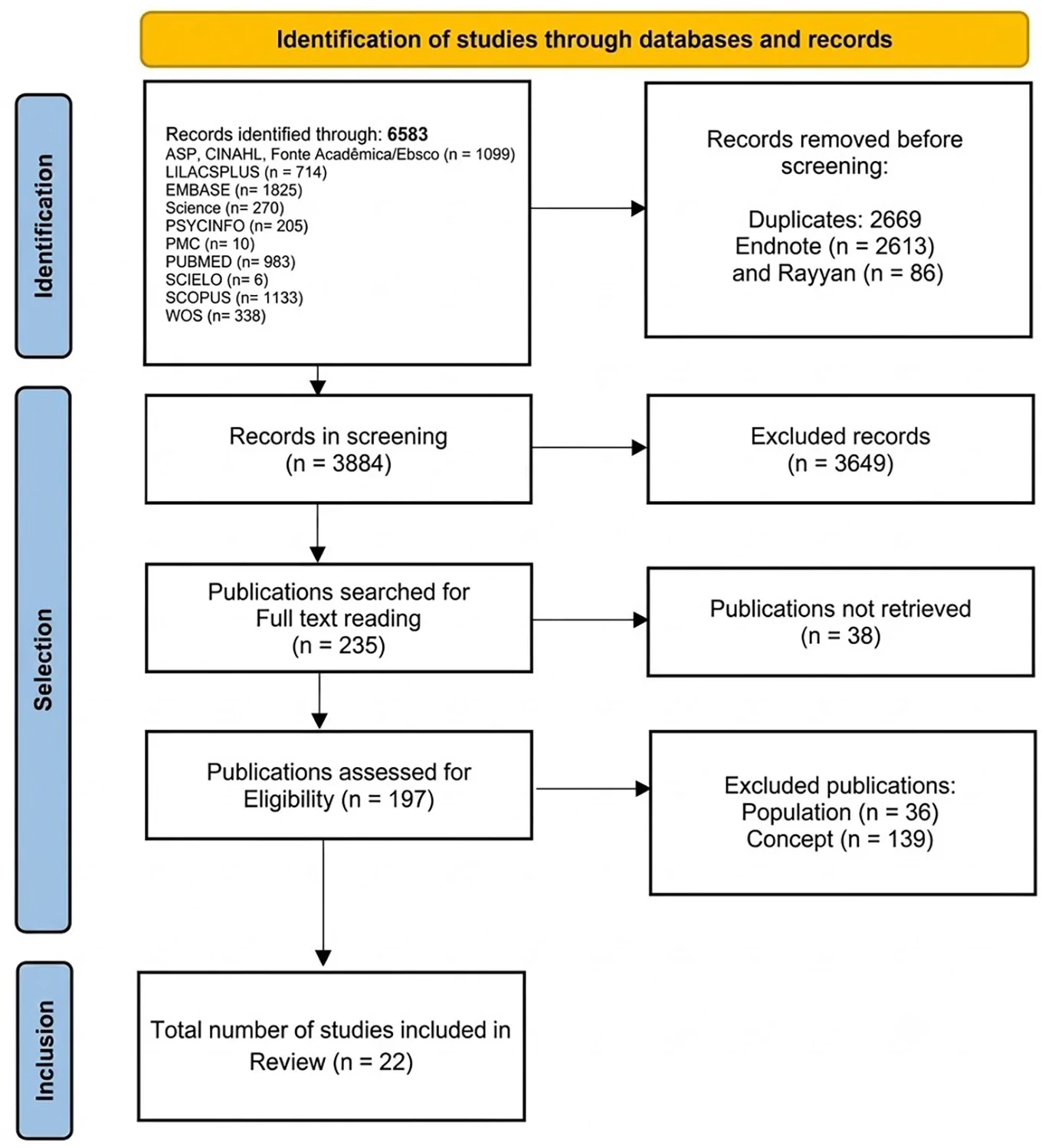
PRISMA-ScR flowchart of the selection process for included studies – Florianópolis, SC, Brazil, 2025.

Based on the application of search strategies in the selected databases and portals, a total of 3,884 were obtained for titles and abstracts reading, of which 235 studies were selected for full-text reading, adhering to the eligibility criteria and the research question. Of these, 22 studies were selected for analysis and discussion, as presented in the PRISMA-ScR flowchart in Figure 1.

Studies started in 1993, with the highest occurrence in 2024 (n = 4; 18%), followed by the year 2021 (n = 3; 14%). Regarding nationality, Canada stands out (n = 6; 27%), followed by the United States (n = 5; 23%). Regarding the type of study, seven (31.5%) consisted of quasi-experimental studies; subsequently, case studies (n = 3; 14%) and participatory qualitative studies (n = 3; 14%). Regarding the setting, the school has a prominent place among the studies included (n = 18, 82%). Some studies included more than one setting, such as rural, urban, and indigenous communities, virtual environments, and universities, among others, as shown in Chart 2.

Regarding the sample/participants, it was observed that the majority of the included studies (n = 18; 82%) were developed with students from public schools. However, some also included teenagers from rural and indigenous communities, as well as teachers, community leaders, young adults, and parents. The sample composition varied according to the type of study and its objectives, which contributed to the diversity of health promotion strategies.

**Figure 2:**
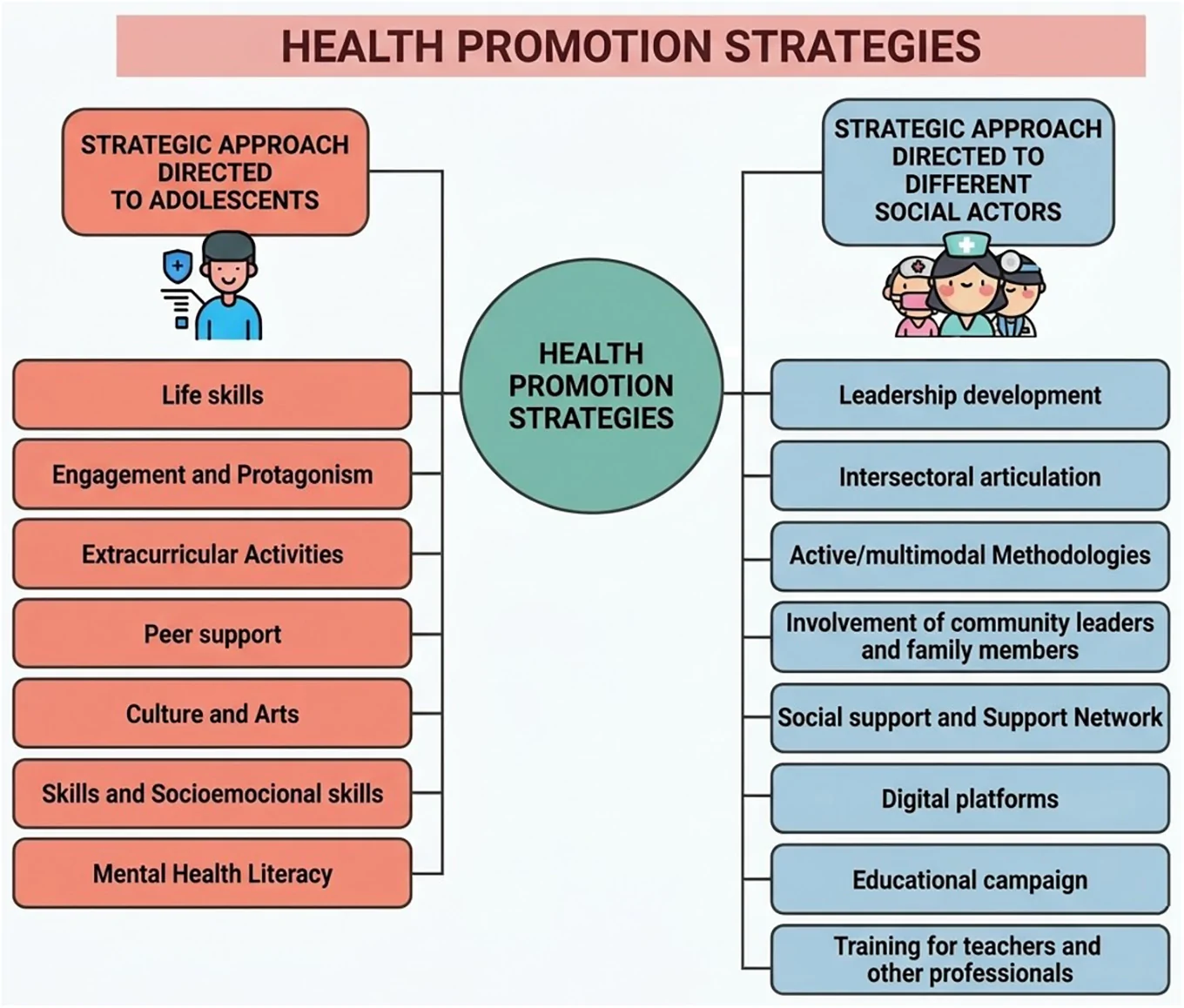
Health promotion strategies targeting adolescents with suicidal behavior – Florianópolis, SC, Brazil, 2025.

Regarding health promotion strategies, two strategic approaches emerged, as illustrated in Figure 2, created using the Google Drawings tool. The identified actions are proposed in a combined and coordinated manner, aiming to broaden the reach and enhance the effects of interventions with adolescents exhibiting suicidal behavior. The first approach is directed at adolescents, highlighting strategies such as developing life skills, peer support, strengthening socio-emotional competencies, mental health literacy, encouraging youth engagement and protagonism, extracurricular activities, and artistic practices. The second approach focuses on different social actors involved in care, including educational campaigns, training for teachers and professionals from various fields, leadership development, involvement of community and family leaders, strengthening of social support and support networks, intersectoral coordination, use of active/multimodal methodologies and digital platforms.

## DISCUSSION

The studies included in this scoping review presented health promotion strategies for adolescents with suicidal behavior, highlighting the school setting, as it is a conducive context for both the implementation of these strategies and suicide prevention^(7,40)^. At school, it is possible to detect changes such as behavioral problems, a drop in academic performance, and frequent absenteeism, which can be early signs of psychological distress in adolescents^(10)^.

Alongside the school environment, the community setting – which includes urban, rural, and indigenous contexts – has gained similar importance in the implementation of health promotion strategies. In these communities, especially when united, the deepening of social networks and participation in collective activities, such as religious practices and group exercise, have shown potential to improve physical and emotional well- being and to reduce the risk of suicide among adolescents^(39,41)^.

Concurrently, digital platforms and social media are emerging as relevant spaces for connecting teenagers with their peers and providing emotional support^(42)^. These tools not only allow the expression of feelings and experiences but also facilitate online interventions, since they promote changes in attitude and encourage people to seek help^(43,44)^.

Primary care services have excelled in school, community, and digital settings, emerging as strategic locations for promoting the mental health of adolescents with suicidal behavior, by expanding access and enabling the implementation of care^(42)^. Although challenges remain, initiatives such as collaborative models and innovative solutions have been developed^(45)^. However, the study points to limitations related to the training and experience of primary care professionals in managing situations such as suicide attempts, which reinforces the need for teams’ continuous training and strengthening^(46)^.

Mapping the intervention scenarios revealed that schools, communities, digital environments, and primary care broaden access and facilitate the implementation of health promotion strategies. These strategies demonstrate diversity and encompass both actions directed at adolescents and initiatives involving the participation of other social actors.

First, the development of life skills and socio-emotional competencies stands out, strengthening autonomy, decision- making, emotion management, resilience, and the building of healthy interpersonal relationships - fundamental elements for promoting mental health and reducing the risk of suicide among adolescents^(18-21,23,29,30,32,36,38)^. To maximize these results, these skills should be worked on in an integrated way within school, family, and health service contexts, with emphasis on the use of the principles of cognitive-behavioral therapy (CBT), recognized for its effectiveness in managing situations related to suicidal behavior and in promoting protective factors^(10,26,36,43,47,48)^.

Other strategies involve strengthening peer support groups, coupled with youth engagement and protagonism, which promote the psychosocial development of adolescents by enabling the sharing of difficult and similar experiences. Furthermore, these initiatives contribute to the improvement of social and relational skills and promote youth empowerment^(28,34,49)^.

In addition to these, other strategies are proving relevant, such as mental health literacy, extracurricular activities, and the use of culture and art, which act as powerful tools for consolidating adolescents’ emotional and community well-being.

Mental health literacy forms an essential basis for promotion and contributes to the adolescents’ overall well-being. Interventions in this area have been shown to improve skills such as self-efficacy, the ability to seek and understand health information, and even reinforce social connections^(7,37)^. Creative strategies, such as the use of theater, were also applied to facilitate mental health literacy in a participatory and contextualized way^(22)^. Another significant point is that culture and art appear as promising strategies and contribute positively to well-being by favoring the expression of emotions, cultural appreciation, and the strengthening of identity pride, especially in indigenous communities^(36)^. Well-structured extracurricular activities, such as sports and music, also stand out as mood regulators and promoters of social bonds, as they reinforce protective factors against psychological distress^(24)^.

The studies focus directly on adolescents, simultaneously highlighting the importance of strategies that broaden the reach of actions, such as educational campaigns, training programs for teachers and health professionals, and the training of community and school leaders, which are fundamental to sustaining health-promoting practices in an integrated and continuous way.

Prevention and awareness campaigns in the media and in public spaces, coordinated at the governmental level, are essential for prevention and promotion, with the potential to influence policies and legislation at all government levels^(47)^. Targeted at rural communities, these actions, combined with policies that expand access to structured activities, enhance youth engagement in health promotion^(21,24)^. As universal strategies, they reach broad audiences and offer essential information to encourage people to seek help^(21)^.

Capacity building, training, and education should be strategically targeted at all professionals and social actors, both within and outside the health sector, with the goal of improving knowledge and skills related to mental health^(47,50)^. These strategies aim, above all, to increase awareness, self-confidence, and the ability of participants to identify suicide risks, intervene early, and provide appropriate support^(22,35,44)^. Evaluations of training programs already implemented demonstrate their effectiveness in promoting care practices and expanding the community response to crisis situations^(31,43)^.

In addition to campaigns, the importance of training and involving community and family leaders is highlighted, focusing on reinforcing adolescents’ trust in their caregivers and repairing ruptures in emotional bonds, factors associated with the risk of suicidal ideation and attempts^(43,44)^. Strategies that encourage family and community engagement, as well as open discussion about suicide, contribute to the creation of welcoming social environments that promote mental health^(7)^. These spaces, by allowing the early observation of signs of distress, become essential in prevention.

Interdisciplinary and intersectoral collaboration between health, education, social assistance, and community organizations enhances health promotion actions and should be encouraged. This integration broadens collective participation in planning and strategy development, thereby consolidating support networks and the effectiveness of interventions^(23,47)^. An intersectoral, territorial, and interdisciplinary approach contributes to bolstering protective factors and, consequently, to reducing suicide rates among adolescents, especially when there is active collaboration within sectors and the community^(26,39,51)^.

Active learning methodologies, such as case studies, role- playing, and problem-based learning, have shown promising results in empowering adolescents to discuss and reflect on suicide, but still require greater implementation and integration into school subject curricula^(10,22,29,37)^. Additionally, innovative strategies such as digital platforms, social media, and apps aimed at teenagers and their families have stood out for facilitating skills learning, expanding support networks, and providing 24-hour online services, establishing themselves as promising resources for promoting mental health^(25,28,33,38)^. However, these initiatives face challenges, such as issues of accessibility, connectivity, data security, the need for financial investment, and cultural adaptations to ensure effectiveness in different contexts^(36,52)^.

Although there are solid regulatory frameworks for the implementation of health promotion policies and strategies in Brazil, institutional articulation, resource availability, technical capacity, and intersectoral coordination still represent significant challenges for their effective implementation within the Brazilian Public Health System (*SUS*)^(53)^. Likewise, studies on mental health promotion indicate that the implementation of effective strategies is hampered by resource limitations, a lack of cost-effectiveness evidence, and contextual barriers that hinder the adaptation of international models to local realities^(54)^.

The strategies identified in this review reinforce the role of nursing in promoting the mental health of adolescents with suicidal behavior. Working in different settings, nurses contribute to expanding access to care, enhancing bonds, and building support networks. These actions require sensitive listening, ongoing training, and intersectoral collaboration, which reaffirms the ethical and political commitment of the profession to the life and well-being of those in distress.

Nevertheless, to support expand these practices, it is necessary to acknowledge the gaps that still exist in scientific production on the subject. Although this scoping review has mapped several health promotion strategies for adolescents with suicidal behavior, it is important to acknowledge the limitations. A significant limitation is the failure to retrieve some of the identified studies, especially the older ones. In total, 38 documents, mostly from the 1980s and 1990s, could not be accessed due to the unavailability of the full text in databases and institutional repositories. Despite conducting additional searches, it was not possible to obtain them, which may have reduced the mapping historical scope.

Moreover, the predominance of studies conducted in high- income countries limits the direct application of these strategies to the Brazilian context, which is marked by specific epidemiological, sociocultural, and structural characteristics. This scenario poses challenges for the development of health promotion policies and practices aimed at adolescents with suicidal behavior and reinforces the need for national and regional research that considers socioeconomic inequalities, social determinants of health, and the organization of public systems, such as the *SUS*. Future research can contribute to the adaptation, validation, and contextualization of international strategies so that they become more sensitive to local realities and culturally appropriate.

## CONCLUSION

The studies analyzed in this review present two strategic approaches that combine and are articulated for prevention and health promotion. The first, directly related to adolescents, aims to boost internal and relational resources through the development of life skills, peer support, socio-emotional competencies, mental health literacy, encouragement of engagement and youth leadership, as well as extracurricular activities and artistic practices. These strategies contribute to the empowerment of adolescents and to fortify their resilience in the face of psychological distress. The second approach is directed at different social actors that make up the broader context of care, such as teachers, health and education professionals, community leaders, and families, who potentiate the creation of care environments and community mobilization, to broaden the reach and effectiveness of health promotion actions through educational campaigns, training for teachers and professionals from various fields, leadership development, social support and support networks, intersectoral articulation, the use of active/multimodal methodologies, and digital platforms.

Furthermore, although the school environment has been widely explored as a space for intervention, there is a need to expand health promotion strategies to other contexts, such as specialized mental health services, to which many adolescents arrive in a critical state of psychological distress and at risk of imminent suicide.

In addition, the need for strategies that go beyond training healthcare professionals through the active incorporation of families in the care process is highlighted. Despite the recognition of their essential role, there is a lack of systematic actions that include them as key players in promoting the health of adolescents with suicidal behavior. This gap underscores the urgent need to develop integrated, intersectoral, and culturally sensitive approaches capable of driving responses to the complex demands of this population and contributing to the reduction of suicide rates among adolescents.

## DATA AVAILABILITY

The data that support the findings of this study are openly available in the Open Science Framework (OSF) at https://doi.org/10.17605/OSF.IO/J9MQB.
