# Supplementary material for: Health promotion strategies for adolescents with suicidal behavior: a scoping review
Source: Rev Esc Enferm USP. 2026 Sep 7;60:e20250338. doi: 10.1590/1980-220X-REEUSP-2025-0338en (PMC13561537; doi:10.1590/1980-220X-REEUSP-2025-0338en)
Supplement: Translation

# Translation: Estratégias de promoção da saúde para adolescentes com comportamento suicida: revisão de escopo

**DOI:** 10.1590/1980-220X-REEUSP-2025-0338pt

**Authors:** Erika Augusta do Amaral Coelho Bezerra, Ivonete Teresinha Schulter Buss Heidemann, Aldalice Aguiar de Souza, Eliana Rosa da Fonseca, Camila Trindade Coelho, Paola Margarita Oñate Daza, Richard Augusto Thomann Beckert, Alaidistânia Aparecida Ferreira

**Affiliations:** 1 Universidade Federal de Santa Catarina, Florianópolis, SC, Brasil.; 2 Universidade do Estado do Amazonas, Manaus, AM, Brasil.; 3 Universidade Federal do Rio de Janeiro, Rio de Janeiro, RJ, Brasil.; 4 Universidade Federal do Amazonas, Manaus, AM, Brasil.

**Keywords:** Adolescente, Estratégias de Saúde, Prevenção do Suicídio, Promoção da Saúde, Suicídio

## Abstract

**Objetivo:**

Mapear estratégias de promoção da saúde a adolescentes com comportamento suicida.

**Método:**

Revisão de escopo conforme diretrizes metodológicas da JBI, sem limitação de idioma e sem recorte temporal. Critérios de inclusão dos estudos: adolescentes de dez a 19 anos com comportamento suicida para explorar o conceito de promoção da saúde, em qualquer serviço de saúde, mas sem limitar esse contexto. Fontes consultadas: MEDLINE/PubMed, PubMed Central/NLM, CINAHL, Academic Search Premier, Fonte Acadêmica/EBSCO, Scopus, Embase/Elsevier, LILACS, BDENF, INDEXPSI, CUMED, BINACIS, LIPECS, MedCarib, BBO, ARGMSAL, BDNPAR, BIGG, BRISA, HomeoIndex, PAHOIRIS, SES-SPBVS, SciELO, PsycInfo/APA.

**Resultados:**

Selecionados 22 artigos, emergiram dois enfoques estratégicos direcionados a adolescentes: desenvolvimento de habilidades para a vida – apoio entre pares, alfabetização em saúde mental, engajamento juvenil – e envolvimento de diferentes atores sociais – família, profissionais, líderes comunitários, redes de apoio intersetoriais.

**Conclusão:**

As estratégias de promoção da saúde foram identificadas e mapeadas em dois enfoques estratégicos: primeiro foram direcionadas aos adolescentes; depois, envolveram diferentes atores sociais ampliando o alcance da promoção da saúde.

## INTRODUÇÃO

As estratégias de promoção da saúde ganham destaque na Atenção Primária à Saúde (APS) pela forte convergência com seus princípios, que favorecem o acesso aos serviços e a implementação de ações integradas^(1)^. A APS também possibilita articular intervenções voltadas tanto para questões individuais quanto coletivas, com o objetivo de melhorar as condições de vida e fortalecer a saúde comunitária^(2,3)^. Nesse sentido, a promoção da saúde (PS) amplia a concepção da saúde e configura-se como uma estratégia promissora para enfrentar os desafios de saúde pública ao olhar sobre os determinantes da saúde e ao potencializar ações intersetoriais^(4,5)^.

Com o propósito de aprimorar as condições de vida e de saúde em dimensões individuais e coletivas, a PS estabelece um leque de estratégias políticas com diretrizes e eixos transversais. Desse modo, as estratégias são caracterizadas pela cooperação entre diferentes setores e dentro do próprio setor de saúde, pela articulação com redes de proteção social e pelo estímulo à ampla participação e ao controle social. Seu principal foco é diminuir vulnerabilidades e riscos resultantes de determinantes multifacetados – sociais, econômicos, políticos, culturais e ambientais. Adicionalmente, prioriza a capacitação da pessoa e da comunidade para um maior controle sobre seu processo de viver. Partindo de uma compreensão abrangente do processo de saúde-doença e de seus condicionantes, a PS busca integrar e valorizar do conhecimento popular ao conhecimento técnico e científico por meio de ações que englobam mudança de comportamento, intervenções coletivas e implementação de políticas públicas integradas^(3,4)^.

Na APS, especialmente no campo da saúde mental, a necessidade de cuidado na perspectiva da PS, orientada pelo paradigma psicossocial, faz com que as pessoas em sofrimento psíquico se tornem sujeitos ativos, inseridos em seus territórios, o que possibilita novas expressões de vida, autonomia e socialização^(6)^.

As pessoas em sofrimento psíquico, principalmente o público infantojuvenil, demandam estratégias amplas e intersetoriais voltadas ao desenvolvimento de habilidades e de competências sociais, fortalecimento, enfrentamento e apoio social, com foco na PS^(4,7)^, considerando sua singularidade e o território em que estão inseridas.

Entre as diversas manifestações de sofrimento psíquico, principalmente entre crianças e adolescentes, o suicídio destaca- se como uma de suas formas mais graves, dada a complexidade e o impacto social, em decorrência da imaturidade neuropsicossocial e emocional desse público. De 2011 a 2022, evidenciou-se um aumento das taxas de suicídio em todas as regiões do Brasil, com maior incidência entre jovens de dez a 24 anos, o que significou um crescimento de 6,14%^(8)^. Na região das Américas, no período de 2000 a 2019, observou-se um aumento de 17% nos casos, enquanto, no Brasil, houve um crescimento expressivo de 43% no número de suicídios^(9)^ nesse mesmo período.

O suicídio entre adolescentes reflete múltiplos fatores contribuintes, como transtornos mentais, conflitos familiares, violência, *bullying*, exclusão social, uso de substâncias como álcool e drogas, além do impacto das tecnologias por meio de mídias e de redes sociais, frequentemente associadas a efeitos negativos sobre a saúde mental^(10,11)^. Diante desse cenário, evidencia-se a necessidade de identificar estratégias não somente restritas à prevenção de agravos, mas também que alcancem o enfoque na PS, que sejam abrangentes e articuladas com diferentes setores capazes de enfrentar a complexidade desses agravos à saúde pública^(10,12)^.

Foram realizadas buscas preliminares com o objetivo de identificar revisões publicadas ou protocolos em andamento com a mesma temática, para garantir a originalidade do estudo e evitar duplicação de esforços. Essa busca incluiu as bases MEDLINE/PubMed, Cochrane Database of Systematic Reviews, Portal Regional da Biblioteca Virtual em Saúde (BVS), PROSPERO e JBI Evidence Synthesis, de modo que se identificaram dois protocolos de revisão: um voltado para estratégias intersetoriais de cuidado a adolescentes com comportamento suicida e autolesão e outro para intervenções destinadas à prevenção e ao manejo do suicídio e da automutilação em contextos educacionais. Embora relacionados ao tema, esses estudos não contemplam o foco específico desta proposta. Assim, não foram encontradas revisões atuais ou em andamento que abordem estratégias de promoção da saúde voltadas a adolescentes com comportamento suicida, o que evidencia uma lacuna no conhecimento e reforça a relevância desta investigação. Portanto, este estudo tem como objetivo mapear estratégias de promoção da saúde direcionadas a adolescentes com comportamento suicida.

## MÉTODO

### Tipo de Estudo

Trata-se de uma revisão de escopo conduzida de acordo com as diretrizes metodológicas do JBI^(13)^, visando assegurar o rigor metodológico, a clareza e a organização do processo de revisão, e relatada conforme orientações do *checklist* PRISMA Extension for Scoping Reviews (PRISMA-ScR)^(14)^. O protocolo desta revisão foi registrado na plataforma Open Science Framework (OSF) sob DOI https://doi.org/10.17605/OSF.IO/J9MQB.

### Questão de Pesquisa

A questão de pesquisa foi definida em conformidade com o acrônimo PCC (população/conceito/contexto), em que a população (P) são “adolescentes com comportamento suicida”; o conceito (C) alude à “promoção da saúde”; e o contexto (C) refere-se a quaisquer serviços de saúde mental em todos os níveis de atenção, mas sem limitação a estes. Estabeleceu-se a seguinte questão de pesquisa: Quais são as estratégias de promoção da saúde para adolescentes com comportamento suicida?

### Critérios de Elegibilidade

População – Esta revisão considera estudos envolvendo adolescentes de dez a 19 anos^(15)^ com comportamento suicida. A Organização Mundial da Saúde (OMS)^(16)^ destaca o suicídio como uma questão grave de saúde pública que requer uma resposta coordenada. Com intervenções oportunas, baseadas em evidências e de baixo custo, é possível reduzir significativamente a ocorrência de suicídios. Embora muitos incidentes sejam impulsivos, o comportamento suicida está fortemente associado a desastres, conflitos, violência, abuso ou perda e sensação de isolamento. A OMS estabelece intervenções eficazes baseadas em evidências, o que inclui a identificação, a avaliação, a gestão e o acompanhamento precoce de qualquer pessoa afetada por comportamentos suicidas^(16)^.

Conceito – Foram incluídos estudos que abordassem estratégias de promoção da saúde para adolescentes com comportamento suicida. Foi considerada a definição da Carta de Ottawa, que define a promoção da saúde como o “processo de capacitação das comunidades para melhorar sua qualidade de vida e saúde, incluindo a participação no controle desse processo”^(4,17)^.

Contexto – Os estudos incluídos foram realizados em qualquer local que oferecesse serviços de saúde mental em qualquer nível de atendimento. Serviços em qualquer nível de assistência médica, levando em conta a faixa etária dos participantes, foram igualmente considerados, mas sem limitação a eles.

Foram considerados estudos com delineamentos experimentais e quase-experimentais (ensaios clínicos randomizados, ensaios controlados não randomizados, estudos antes e depois e séries temporais interrompidas), observacionais analíticos (coortes prospectivas e retrospectivas, caso-controle e transversais analíticos) e observacionais descritivos (séries de casos, relatos de caso e estudos transversais descritivos). Foram incluídos estudos qualitativos (fenomenologia, teoria fundamentada, etnografia, descrição qualitativa e pesquisa-ação), além de textos teóricos, artigos de opinião, dissertações e teses. Foram excluídas revisões sistemáticas, de escopo e integrativas, bem como resumos de eventos, cartas e editoriais.

### Estratégia de Busca

A estratégia de busca foi realizada em três etapas, visando localizar estudos publicados e não publicados, em colaboração com uma bibliotecária especializada, certificada pelo JBI em métodos de revisão. Inicialmente, foi conduzida uma busca preliminar nos portais – MEDLINE/PubMed, Cochrane Database of Systematic Reviews, Portal Regional da Biblioteca Virtual em Saúde (BVS), PROSPERO e JBI Evidence Synthesis. Na segunda etapa, a estratégia definitiva apresentada no Quadro 1 foi adaptada para as bases de dados/portais: MEDLINE/PubMed, PubMed Central/NLM, CINAHL, Academic Search Premier, Fonte Acadêmica/EBSCO, Scopus, Embase/Elsevier, LILACS, BDENF, INDEXPSI, CUMED, BINACIS, LIPECS, MedCarib, BBO, ARGMSAL, BDNPAR, BIGG, BRISA, HomeoIndex, PAHOIRIS, SES-SPLILACS, BDENF, Index Psicologia/BVS, SciELO e PsycInfo/APA. A literatura cinzenta foi explorada pela plataforma Science.gov; e acrescentou-se a ferramenta de busca Google Scholar. Não houve restrição de idioma ou período de publicação. Por fim, na terceira etapa, foi realizada a busca manual nas referências dos estudos incluídos. A busca foi feita em 23 de janeiro de 2025.

**Quadro 1:** Estratégias de busca para as bases de dados/portais – Florianópolis, SC, Brasil, 2025.

Bases de dados/portais	Estratégias	Total
BVS	((adolescente OR adolescência OR adolescente* OR jovem OR jovens OR juventude OR joven OR jóvenes OR juventud OR jeunes OR jeunesse OR adolescent OR adolescence OR adolescent* OR teen* OR teenager* OR youth*) AND (“Ideação Suicida” OR “Ideación Suicida” OR “Ideas Suicidas” OR “Idéation suicidaire” OR “Idéation de suicide” OR “Idées suicidaires” OR “Comportamento Autodestrutivo” OR “Autoagressão Intencional” OR autolesão OR “Autolesão Intencional” OR “Autolesão não Suicida” OR “Conduta Autolesiva” OR “Ferimento Autoinfligido não Suicida” OR “Lesão Autoinfligida não Suicida” OR “Conducta Autodestructiva” OR “Autolesión Intencional” OR “Autolesión no Suicida” OR autolesiones OR “Herimiento Autoinferido no Suicida” OR “Herimientos Autoinferidos” OR “Comportement auto-agressif” OR “Automutilation délibérée” OR “Automutilation non suicidaire” OR “Comportement autoagressif” OR “Comportement autodestructeur” OR “Comportement d’auto-agression” OR “Comportement d’autoagression” OR “Comportement d’automutilation” OR suicídio OR suicídio* OR suicide OR “Suicidal Ideation” OR “Suicidal Ideations” OR “Self-Injurious Behavior” OR “Deliberate Self Harm” OR “Deliberate Self-Harm” OR “Intentional Self Harm” OR “Intentional Self Injuries” OR “Intentional Self Injury” OR “Non Suicidal Self Injury” OR “Non-Suicidal Self Injuries” OR “Non-Suicidal Self Injury” OR “Nonsuicidal Self Injuries” OR “Nonsuicidal Self Injury” OR “Self Destructive Behavior” OR “Self Harm” OR “Self Injurious Behavior” OR “Self Injury” OR “Self-Destructive Behavior” OR “Self-Destructive Behaviors” OR self-injuries OR “Self-Injurious Behaviors” OR self-injury OR suicide OR suicide* OR “Prevenção do Suicídio” OR “Conscientização sobre Suicídio” OR “Prevenção contra Suicídio” OR “Prevención del Suicidio” OR “Conciencia Sobre el Suicidio” OR “Prévention du suicide” OR “Suicide Prevention” OR “Suicide Awareness” OR “Suicide Preventions”)) AND ((“Promoção da Saúde” OR “Ambientes Apoiadores de Saúde” OR “Ambientes de Apoio à Saúde” OR “Campanhas de Saúde” OR “Item Promocional” OR “Itens Promocionais” OR “Programas de Bem-Estar” OR “Promoção do Bem Estar” OR “Promoção em Saúde” OR “Promoción de la Salud” OR “Ambientes de Apoyo a la Salud” OR “Campanas de Salud” OR “Entornos Apoyadores de la Salud” OR “Entornos de Apoyo a la Salud” OR “Items Promocionales” OR “Programas de Bienestar” OR “Promoción del Bienestar” OR “Promotion de la santé” OR “Campagnes de santé” OR “Campagnes pour la santé” OR “Points promotionnels” OR “Programmes de bien-être” OR “pessoas saudáveis” OR “programas para pessoas saudáveis” OR “Éducation Environnementale” OR “Educação em Saúde” OR “Educar para a Saúde” OR “Educação para a Saúde” OR “Educação Sanitária” OR “Educación en Salud” OR “Educación para la Salud” OR “Educación para la Salud Comunitaria” OR “Educación Sanitaria” OR “Éducation pour la santé” OR “Éducation communautaire en santé” OR “Éducation en santé” OR “Éducation pour la santé au sein de la communauté” OR “Éducation sanitaire” OR “Éducation sanitaire communautaire” OR “Formation communautaire en santé” OR “Health Promotion” OR “Health Campaign” OR “Health Campaigns” OR “Health Promotions” OR “Health-Supportive Environments” OR “Promotion of Health” OR “Promotional Item” OR “Promotional Items” OR “Wellness Program” OR “Wellness Programs” OR “healthy people 2010”or “healthy people programmes” OR “healthy people programs” OR “Health Education” OR “Community Health Education”)) AND db:(“LILACS” OR “BDENF” OR “INDEXPSI” OR “CUMED” OR “BINACIS” OR “LIPECS” OR “MedCarib” OR “BBO” OR “ARGMSAL” OR “BDNPAR” OR “BIGG” OR “BRISA” OR “HomeoIndex” OR “PAHOIRIS” OR “SES-SP”) AND instance:”lilacsplus”	714
Academic Searche Premier ( ) CINAHL with Full Text Fonte Acadêmica ( )	((TI Adolescent OR Adolescence OR Adolescent* OR Teen* OR Teenager* OR Youth* OR SU Adolescent OR Adolescence OR Adolescent* OR Teen* OR Teenager* OR Youth* OR AB Adolescent OR Adolescence OR Adolescent* OR Teen* OR Teenager* OR Youth*) AND (TI “Suicidal Ideation” OR “Suicidal Ideations” OR “Self-Injurious Behavior” OR “Deliberate Self Harm” OR “Deliberate Self-Harm” OR “Intentional Self Harm” OR “Intentional Self Injuries” OR “Intentional Self Injury” OR “Non Suicidal Self Injury” OR “Non-Suicidal Self Injuries” OR “Non-Suicidal Self Injury” OR “Nonsuicidal Self Injuries” OR “Nonsuicidal Self Injury” OR “Self Destructive Behavior” OR “Self Harm” OR “Self Injurious Behavior” OR “Self Injury” OR “Self-Destructive Behavior” OR “Self-Destructive Behaviors” OR Self-Injuries OR “Self-Injurious Behaviors” OR Self-Injury OR Suicide OR Suicide* OR “Suicide Prevention” OR “Awareness, Suicide” OR “Prevention, Suicide” OR “Suicide Awareness” OR “Suicide Preventions” OR SU “Suicidal Ideation” OR “Suicidal Ideations” OR “Self-Injurious Behavior” OR “Deliberate Self Harm” OR “Deliberate Self-Harm” OR “Intentional Self Harm” OR “Intentional Self Injuries” OR “Intentional Self Injury” OR “Non Suicidal Self Injury” OR “Non-Suicidal Self Injuries” OR “Non-Suicidal Self Injury” OR “Nonsuicidal Self Injuries” OR “Nonsuicidal Self Injury” OR “Self Destructive Behavior” OR “Self Harm” OR “Self Injurious Behavior” OR “Self Injury” OR “Self-Destructive Behavior” OR “Self-Destructive Behaviors” OR Self-Injuries OR “Self-Injurious Behaviors” OR Self-Injury OR Suicide OR Suicide* OR “Suicide Prevention” OR “Awareness, Suicide” OR “Prevention, Suicide” OR “Suicide Awareness” OR “Suicide Preventions” OR AB “Suicidal Ideation” OR “Suicidal Ideations” OR “Self-Injurious Behavior” OR “Deliberate Self Harm” OR “Deliberate Self-Harm” OR “Intentional Self Harm” OR “Intentional Self Injuries” OR “Intentional Self Injury” OR “Non Suicidal Self Injury” OR “Non-Suicidal Self Injuries” OR “Non-Suicidal Self Injury” OR “Nonsuicidal Self Injuries” OR “Nonsuicidal Self Injury” OR “Self Destructive Behavior” OR “Self Harm” OR “Self Injurious Behavior” OR “Self Injury” OR “Self-Destructive Behavior” OR “Self-Destructive Behaviors” OR Self-Injuries OR “Self-Injurious Behaviors” OR Self-Injury OR Suicide OR Suicide* OR “Suicide Prevention” OR “Awareness, Suicide” OR “Prevention, Suicide” OR “Suicide Awareness” OR “Suicide Preventions”)) AND (TI “Health Promotion” OR “Health Campaign” OR “Health Campaigns” OR “Health Promotions” OR “Health-Supportive Environments” OR “Promotion of Health” OR “Promotional Item” OR “Promotional Items” OR “Wellness Program” OR “Wellness Programs” OR “healthy people 2010”OR “healthy people programmes” OR “healthy people programs” OR “Health Education” OR “Community Health Education” OR SU “Health Promotion” OR “Health Campaign” OR “Health Campaigns” OR “Health Promotions” OR “Health-Supportive Environments” OR “Promotion of Health” OR “Promotional Item” OR “Promotional Items” OR “Wellness Program” OR “Wellness Programs” OR “healthy people 2010”OR “healthy people programmes” OR “healthy people programs” OR “Health Education” OR “Community Health Education” OR AB “Health Promotion” OR “Health Campaign” OR “Health Campaigns” OR “Health Promotions” OR “Health-Supportive Environments” OR “Promotion of Health” OR “Promotional Item” OR “Promotional Items” OR “Wellness Program” OR “Wellness Programs” OR “healthy people 2010”OR “healthy people programmes” OR “healthy people programs” OR “Health Education” OR “Community Health Education”)	1099
EMBASE	(‘adolescent’/exp OR adolescent OR ‘adolescence’/exp OR adolescence OR adolescent* OR teen* OR teenager* OR youth*) AND (‘suicidal ideation’/exp OR ‘suicidal ideation’ OR ‘suicidal ideations’ OR ‘self-injurious behavior’/exp OR ‘self-injurious behavior’ OR ‘deliberate self harm’/exp OR ‘deliberate self harm’ OR ‘deliberate self-harm’/exp OR ‘deliberate self-harm’ OR ‘intentional self harm’ OR ‘intentional self injuries’ OR ‘intentional self injury’ OR ‘non suicidal self injury’/exp OR ‘non suicidal self injury’ OR ‘non-suicidal self injuries’ OR ‘non-suicidal self injury’/exp OR ‘non-suicidal self injury’ OR ‘nonsuicidal self injuries’ OR ‘nonsuicidal self injury’/exp OR ‘nonsuicidal self injury’ OR ‘self destructive behavior’/exp OR ‘self destructive behavior’ OR ‘self harm’/exp OR ‘self harm’ OR ‘self injurious behavior’/exp OR ‘self injurious behavior’ OR ‘self-destructive behavior’ OR ‘self-destructive behaviors’ OR ‘self injuries’ OR ‘self-injurious behaviors’ OR ‘self injury’/exp OR ‘self injury’ OR ‘suicide’/exp OR suicide OR suicide* OR ‘suicide prevention’/exp OR ‘suicide prevention’ OR ‘awareness, suicide’ OR ‘prevention, suicide’ OR ‘suicide awareness’ OR ‘suicide preventions’) AND (‘health promotion’/exp OR ‘health promotion’ OR ‘health campaign’ OR ‘health campaigns’ OR ‘health promotions’ OR ‘health-supportive environments’ OR ‘promotion of health’ OR ‘promotional item’ OR ‘promotional items’/exp OR ‘promotional items’ OR ‘wellness program’ OR ‘wellness programs’ OR ‘healthy people 2010’/exp OR ‘healthy people 2010’ OR ‘healthy people programmes’/exp OR ‘healthy people programmes’ OR ‘healthy people programs’/exp OR ‘healthy people programs’ OR ‘health education’/exp OR ‘health education’ OR ‘community health education’) AND ([article]/lim OR [article in press]/lim OR [conference paper]/lim OR [conference review]/lim OR [review]/lim OR [preprint]/lim)	1825
Science	Title: (adolescent OR adolescence OR adolescent* OR teen* OR teenager* OR youth*) AND (“Suicidal Ideation” OR “Suicidal Ideations” OR “Self-Injurious Behavior” OR “Deliberate Self Harm” OR “Deliberate Self-Harm” OR “Intentional Self Harm” OR “Intentional Self Injuries” OR “Intentional Self Injury” OR “Non Suicidal Self Injury” OR “Non-Suicidal Self Injuries” OR “Non-Suicidal Self Injury” OR “Nonsuicidal Self Injuries” OR “Nonsuicidal Self Injury” OR “Self Destructive Behavior” OR “Self Harm” OR “Self Injurious Behavior” OR “Self Injury” OR “Self-Destructive Behavior” OR “Self-Destructive Behaviors” OR self-injuries OR “Self-Injurious Behaviors” OR self-injury OR suicide OR suicide* OR “Suicide Prevention” OR “Awareness, Suicide” OR “Prevention, Suicide” OR “Suicide Awareness” OR “Suicide Preventions”) AND (“Health Promotion” OR “Health Campaign” OR “Health Campaigns” OR “Health Promotions” OR “Health-Supportive Environments” OR “Promotion of Health” OR “Promotional Item” OR “Promotional Items” OR “Wellness Program” OR “Wellness Programs” OR “healthy people 2010” OR “healthy people programmes” OR “healthy people programs” OR “Health Education” OR “Community Health Education”)	270
PSYCINFO	(Title: adolescent OR Title: adolescence OR Title: adolescent* OR Title: teen* OR Title: teenager* OR Title: youth*) AND (Title: “Suicidal Ideation” OR Title: “Suicidal Ideations” OR Title: “Self-Injurious Behavior” OR Title: “Deliberate Self Harm” OR Title: “Deliberate Self-Harm” OR Title: “Intentional Self Harm” OR Title: “Intentional Self Injuries” OR Title: “Intentional Self Injury” OR Title: “Non Suicidal Self Injury” OR Title: “Non-Suicidal Self Injuries” OR Title: “Non-Suicidal Self Injury” OR Title: “Nonsuicidal Self Injuries” OR Title: “Nonsuicidal Self Injury” OR Title: “Self Destructive Behavior” OR Title: “Self Harm” OR Title: “Self Injurious Behavior” OR Title: “Self Injury” OR Title: “Self-Destructive Behavior” OR Title: “Self-Destructive Behaviors” OR Title: self-injuries OR Title: “Self-Injurious Behaviors” OR Title: self-injury OR Title: suicide OR Title: suicide* OR Title: “Suicide Prevention” OR Title: “Awareness, Suicide” OR Title: “Prevention, Suicide” OR Title: “Suicide Awareness” OR Title: “Suicide Preventions”) AND (Title: “Health Promotion” OR Title: “Health Campaign” OR Title: “Health Campaigns” OR Title: “Health Promotions” OR Title: “Health-Supportive Environments” OR Title: “Promotion of Health” OR Title: “Promotional Item” OR Title: “Promotional Items” OR Title: “Wellness Program” OR Title: “Wellness Programs” OR Title: “healthy people 2010” OR Title: “healthy people programmes” OR Title: “healthy people programs” OR Title: “Health Education” OR Title: “Community Health Education”) OR (Abstract: adolescent OR Abstract: adolescence OR Abstract: adolescent* OR Abstract: teen* OR Abstract: teenager* OR Abstract: youth*) AND (Abstract: “Suicidal Ideation” OR Abstract: “Suicidal Ideations” OR Abstract: “Self-Injurious Behavior” OR Abstract: “Deliberate Self Harm” OR Abstract: “Deliberate Self-Harm” OR Abstract: “Intentional Self Harm” OR Abstract: “Intentional Self Injuries” OR Abstract: “Intentional Self Injury” OR Abstract: “Non Suicidal Self Injury” OR Abstract: “Non-Suicidal Self Injuries” OR Abstract: “Non-Suicidal Self Injury” OR Abstract: “Nonsuicidal Self Injuries” OR Abstract: “Nonsuicidal Self Injury” OR Abstract: “Self Destructive Behavior” OR Abstract: “Self Harm” OR Abstract: “Self Injurious Behavior” OR Abstract: “Self Injury” OR Abstract: “Self-Destructive Behavior” OR Abstract: “Self-Destructive Behaviors” OR Abstract: self-injuries OR Abstract: “Self-Injurious Behaviors” OR Abstract: self-injury OR Abstract: suicide OR Abstract: suicide* OR Abstract: “Suicide Prevention” OR Abstract: “Awareness, Suicide” OR Abstract: “Prevention, Suicide” OR Abstract: “Suicide Awareness” OR Abstract: “Suicide Preventions”) AND (Abstract: “Health Promotion” OR Abstract: “Health Campaign” OR Abstract: “Health Campaigns” OR Abstract: “Health Promotions” OR Abstract: “Health-Supportive Environments” OR Abstract: “Promotion of Health” OR Abstract: “Promotional Item” OR Abstract: “Promotional Items” OR Abstract: “Wellness Program” OR Abstract: “Wellness Programs” OR Abstract: “healthy people 2010” OR Abstract: “healthy people programmes” OR Abstract: “healthy people programs” OR Abstract: “Health Education” OR Abstract: “Community Health Education”) OR (Keywords: adolescent OR Keywords: adolescence OR Keywords: adolescent* OR Keywords: teen* OR Keywords: teenager* OR Keywords: youth*) AND (Keywords: “Suicidal Ideation” OR Keywords: “Suicidal Ideations” OR Keywords: “Self-Injurious Behavior” OR Keywords: “Deliberate Self Harm” OR Keywords: “Deliberate Self-Harm” OR Keywords: “Intentional Self Harm” OR Keywords: “Intentional Self Injuries” OR Keywords: “Intentional Self Injury” OR Keywords: “Non Suicidal Self Injury” OR Keywords: “Non-Suicidal Self Injuries” OR Keywords: “Non-Suicidal Self Injury” OR Keywords: “Nonsuicidal Self Injuries” OR Keywords: “Nonsuicidal Self Injury” OR Keywords: “Self Destructive Behavior” OR Keywords: “Self Harm” OR Keywords: “Self Injurious Behavior” OR Keywords: “Self Injury” OR Keywords: “Self-Destructive Behavior” OR Keywords: “Self-Destructive Behaviors” OR Keywords: self-injuries OR Keywords: “Self-Injurious Behaviors” OR Keywords: self-injury OR Keywords: suicide OR Keywords: suicide* OR Keywords: “Suicide Prevention” OR Keywords: “Awareness, Suicide” OR Keywords: “Prevention, Suicide” OR Keywords: “Suicide Awareness” OR Keywords: “Suicide Preventions”) AND (Keywords: “Health Promotion” OR Keywords: “Health Campaign” OR Keywords: “Health Campaigns” OR Keywords: “Health Promotions” OR Keywords: “Health-Supportive Environments” OR Keywords: “Promotion of Health” OR Keywords: “Promotional Item” OR Keywords: “Promotional Items” OR Keywords: “Wellness Program” OR Keywords: “Wellness Programs” OR Keywords: “healthy people 2010” OR Keywords: “healthy people programmes” OR Keywords: “healthy people programs” OR Keywords: “Health Education” OR Keywords: “Community Health Education”)	205
PMC	(Title: (adolescent[Abstract] OR adolescence[Abstract] OR adolescent*[Abstract] OR teen*[Abstract] OR teenager*[Abstract] OR youth*)[Abstract] AND (“Suicidal Ideation”[Abstract] OR “Suicidal Ideations”[Abstract] OR “Self-Injurious Behavior”[Abstract] OR “Deliberate Self Harm”[Abstract] OR “Deliberate Self-Harm”[Abstract] OR “Intentional Self Harm”[Abstract] OR “Intentional Self Injuries”[Abstract] OR “Intentional Self Injury”[Abstract] OR “Non Suicidal Self Injury”[Abstract] OR “Non-Suicidal Self Injuries”[Abstract] OR “Non-Suicidal Self Injury”[Abstract] OR “Nonsuicidal Self Injuries”[Abstract] OR “Nonsuicidal Self Injury”[Abstract] OR “Self Destructive Behavior”[Abstract] OR “Self Harm”[Abstract] OR “Self Injurious Behavior”[Abstract] OR “Self Injury”[Abstract] OR “Self-Destructive Behavior”[Abstract] OR “Self-Destructive Behaviors”[Abstract] OR self-injuries[Abstract] OR “Self-Injurious Behaviors”[Abstract] OR self-injury[Abstract] OR suicide[Abstract] OR suicide*[Abstract] OR “Suicide Prevention”[Abstract] OR “Awareness, Suicide”[Abstract] OR “Prevention, Suicide”[Abstract] OR “Suicide Awareness”[Abstract] OR “Suicide Preventions”)[Abstract] AND (“Health Promotion”[Abstract] OR “Health Campaign”[Abstract] OR “Health Campaigns”[Abstract] OR “Health Promotions”[Abstract] OR “Health-Supportive Environments”[Abstract] OR “Promotion of Health”[Abstract] OR “Promotional Item”[Abstract] OR “Promotional Items”[Abstract] OR “Wellness Program”[Abstract] OR “Wellness Programs”[Abstract] OR “healthy people 2010”[Abstract] OR “healthy people programmes”[Abstract] OR “healthy people programs”[Abstract] OR “Health Education”[Abstract] OR “Community Health Education”)[Abstract])	10
PUBMED	(Adolescent[mh] OR Adolescence OR Adolescent* OR Teen* OR Teenager* OR Youth*) AND (“Suicidal Ideation”[mh] OR “Suicidal Ideations” OR “Self-Injurious Behavior”[mh] OR “Deliberate Self Harm” OR “Deliberate Self-Harm” OR “Intentional Self Harm” OR “Intentional Self Injuries” OR “Intentional Self Injury” OR “Non Suicidal Self Injury” OR “Non-Suicidal Self Injuries” OR “Non-Suicidal Self Injury” OR “Nonsuicidal Self Injuries” OR “Nonsuicidal Self Injury” OR “Self Destructive Behavior” OR “Self Harm” OR “Self Injurious Behavior” OR “Self Injury” OR “Self-Destructive Behavior” OR “Self-Destructive Behaviors” OR Self-Injuries OR “Self-Injurious Behaviors” OR Self-Injury OR Suicide[mh] OR Suicide* OR “Suicide Prevention”[mh] OR “Suicide Awareness” OR “Suicide Preventions”) AND (“Health Promotion”[mh] OR Health Campaign*[tiab] OR Health Promotion*[tiab] OR “Health-Supportive Environments”[tiab] OR “Promotion of Health”[tiab] OR Promotional Item*[tiab] OR Wellness Program*[tiab] OR “healthy people”[tiab] OR healthy people program*[tiab] OR “Health Education”[mh] OR Health Education[tiab])	983
SCIELO	(adolescent OR adolescence OR adolescent* OR teen* OR teenager* OR youth*) AND (“Suicidal Ideation” OR “Suicidal Ideations” OR “Self-Injurious Behavior” OR “Deliberate Self Harm” OR “Deliberate Self-Harm” OR “Intentional Self Harm” OR “Intentional Self Injuries” OR “Intentional Self Injury” OR “Non Suicidal Self Injury” OR “Non-Suicidal Self Injuries” OR “Non-Suicidal Self Injury” OR “Nonsuicidal Self Injuries” OR “Nonsuicidal Self Injury” OR “Self Destructive Behavior” OR “Self Harm” OR “Self Injurious Behavior” OR “Self Injury” OR “Self-Destructive Behavior” OR “Self-Destructive Behaviors” OR self-injuries OR “Self-Injurious Behaviors” OR self-injury OR suicide OR suicide* OR “Suicide Prevention” OR “Awareness, Suicide” OR “Prevention, Suicide” OR “Suicide Awareness” OR “Suicide Preventions”) AND (“Health Promotion” OR “Health Campaign” OR “Health Campaigns” OR “Health Promotions” OR “Health-Supportive Environments” OR “Promotion of Health” OR “Promotional Item” OR “Promotional Items” OR “Wellness Program” OR “Wellness Programs” OR “healthy people 2010” OR “healthy people programmes” OR “healthy people programs” OR “Health Education” OR “Community Health Education”)	6
SCOPUS	(TITLE-ABS-KEY (adolescent OR adolescence OR adolescent* OR teen* OR teenager* OR youth*) AND TITLE-ABS-KEY (“Suicidal Ideation” OR “Suicidal Ideations” OR “Self-Injurious Behavior” OR “Deliberate Self Harm” OR “Deliberate Self-Harm” OR “Intentional Self Harm” OR “Intentional Self Injuries” OR “Intentional Self Injury” OR “Non Suicidal Self Injury” OR “Non-Suicidal Self Injuries” OR “Non-Suicidal Self Injury” OR “Nonsuicidal Self Injuries” OR “Nonsuicidal Self Injury” OR “Self Destructive Behavior” OR “Self Harm” OR “Self Injurious Behavior” OR “Self Injury” OR “Self-Destructive Behavior” OR “Self-Destructive Behaviors” OR self-injuries OR “Self-Injurious Behaviors” OR self-injury OR suicide OR suicide* OR “Suicide Prevention” OR “Awareness, Suicide” OR “Prevention, Suicide” OR “Suicide Awareness” OR “Suicide Preventions”) AND TITLE-ABS-KEY (“Health Promotion” OR “Health Campaign” OR “Health Campaigns” OR “Health Promotions” OR “Health-Supportive Environments” OR “Promotion of Health” OR “Promotional Item” OR “Promotional Items” OR “Wellness Program” OR “Wellness Programs” OR “healthy people 2010” OR “healthy people programmes” OR “healthy people programs” OR “Health Education” OR “Community Health Education”))	1133
WOS	TS=(Adolescent OR Adolescence OR Adolescent* OR Teen* OR Teenager* OR Youth*) AND TS=(“Suicidal Ideation” OR “Suicidal Ideations” OR “Self-Injurious Behavior” OR “Deliberate Self Harm” OR “Deliberate Self-Harm” OR “Intentional Self Harm” OR “Intentional Self Injuries” OR “Intentional Self Injury” OR “Non Suicidal Self Injury” OR “Non-Suicidal Self Injuries” OR “Non-Suicidal Self Injury” OR “Nonsuicidal Self Injuries” OR “Nonsuicidal Self Injury” OR “Self Destructive Behavior” OR “Self Harm” OR “Self Injurious Behavior” OR “Self Injury” OR “Self-Destructive Behavior” OR “Self-Destructive Behaviors” OR Self-Injuries OR “Self-Injurious Behaviors” OR Self-Injury OR Suicide OR Suicide* OR “Suicide Prevention” OR “Awareness, Suicide” OR “Prevention, Suicide” OR “Suicide Awareness” OR “Suicide Preventions”) AND TS=(“Health Promotion” OR “Health Campaign” OR “Health Campaigns” OR “Health Promotions” OR “Health-Supportive Environments” OR “Promotion of Health” OR “Promotional Item” OR “Promotional Items” OR “Wellness Program” OR “Wellness Programs” OR “healthy people 2010” OR “healthy people programmes” OR “healthy people programs” OR “Health Education” OR “Community Health Education”)	338
TOTAL		6583

### Seleção dos Estudos

Após a busca nas bases de dados, as referências identificadas foram carregadas para o EndNote 21 (Clarivate Analytics, PA, EUA), e duplicatas foram removidas. Em seguida, essas referências foram exportadas para o *software* Intelligent Systematic Review (Rayyan) para o gerenciamento do processo de seleção. Os documentos foram avaliados por título e por resumo a partir dos critérios de inclusão, processo conduzido por quatro revisores independentes, feito após um teste piloto. Após avaliação, as fontes relevantes foram recuperadas e avaliadas na íntegra, e as decisões foram marcadas no Rayyan.

As divergências que surgiram entre os revisores em cada etapa do processo de seleção foram resolvidas por meio de discussão, de consenso e ainda de um quinto revisor. Os resultados da busca e do processo de inclusão dos estudos estão apresentados no diagrama de fluxo, conforme as diretrizes do PRISMA-ScR^(14)^.

### Extração e Análise dos Dados

Os dados foram extraídos dos artigos incluídos por quatro revisores independentes por meio da utilização de uma ferramenta de extração de dados (Quadro 2) desenvolvida pelos revisores, a partir do manual da JBI. Os dados incluíram detalhes específicos sobre as características dos estudos (citação, ano, país, objetivo, tipo de estudo, amostra), participantes, conceito, contexto (PCC), métodos de estudo e principais conclusões relevantes para a questão de revisão.

**Quadro 2:** Caracterização dos estudos incluídos na revisão de escopo – Florianópolis, SC, Brasil, 2025.

Citação/ano/país		Objetivo	Método/Tipo de estudo	Amostra/participantes	Cenário de intervenção/contexto	Principais resultados/estratégias
1	Klingman e Hochdorf^(18)^, 1993, Israel.	Avaliar a eficácia de um programa escolar de prevenção primária e a capacitação de alunos como guardiões frente a comportamentos autodestrutivos.	Estudo quase-experimental com grupo de intervenção e grupo controle	237 alunos da 8ª série (12 a 13 anos)	Escola pública	Habilidade para vida Apoio entre pares
2	Eggert et al.^(19)^, 1994, Estados Unidos.	Avaliar a eficácia de um programa escolar de prevenção primária voltado para a redução do risco de suicídio e do uso de drogas.	Estudo quase-experimental com grupo de intervenção e grupo controle	Cerca de 1.000 alunos de cinco escolas de ensino médio	Escola pública	Habilidade para vida Atividades extracurriculares Apoio entre pares
3	LaFromboise e Howard-Pitney^(20)^, 1995, Estados Unidos.	Avaliar a eficácia de um programa de prevenção de suicídio com foco em habilidades para a vida.	Estudo quase-experimental com grupo de intervenção e grupo controle	128 alunos do ensino médio (14 a 19 anos)	Escola pública	Habilidade para vida
4	Coggan et al.^(21)^, 1997, Nova Zelândia.	Investigar as opiniões dos jovens sobre o suicídio entre jovens	Estudo qualitativo (grupos focais)	140 jovens entre 16 e 24 anos	Comunidade, escolas, universidades	Habilidade para vida Treinamento para professores e outros profissionais Campanha educativa Engajamento e protagonismo Apoio social e rede de apoio
5	Waring et al.^(22)^, 2000, Austrália.	Descrever vários projetos do Hunter Institute of Mental Health, com foco na promoção e prevenção da saúde mental em adolescentes	Relato de experiência / Descritivo.	Não há amostra de participantes específica, mas inclui descrição de projetos voltados a estudantes do ensino médio e universitários	Ambientes escolares (ensino médio e universidades), serviços de saúde, comunidade geral.	Alfabetização em saúde mental Apoio social e rede de apoio Treinamento para professores e outros profissionais Cultura e arte Metodologia ativa/multimodal
6	Day^(23)^, 2004, Reino Unido.	Investigar se o ensino de habilidades de resolução de problemas para adolescentes pode ser uma estratégia eficaz na prevenção do suicídio juvenil.	Pesquisa-ação com abordagem qualitativa e quantitativa.	Alunos de 11 a 12 anos do 7º ano e professores, enfermeiros escolares e profissionais de saúde mental	Ambiente escolar	Habilidade para vida Articulação intersetorial
7	Armstrong^(24)^, 2011, Canadá.	Analisar os fatores de risco e proteção associados à ideação suicida em adolescentes de contextos rurais e urbanos e propor estratégias de intervenção.	Estudo quantitativo, transversal, descritivo e analítico.	813 alunos do ensino médio: 459 de áreas rurais e 354 de áreas urbanas	Ambiente escolar e comunitário de zonas rurais e urbanas	Engajamento e protagonismo Atividades extracurriculares
8	Carli^(25)^, 2016, Suíça.	Desenvolver e avaliar a efetividade de uma intervenção *online* de promoção da saúde mental e prevenção do suicídio, voltada para adolescentes europeus, por meio do projeto SUPREME.	Ensaio clínico randomizado, multicêntrico, simples-cego.	2.286 alunos, de 14 a 18 anos de seis países europeus: Suécia, Itália, Inglaterra, Espanha, Lituânia, Estônia e Hungria.	Ambiente escolar e *online* (plataforma virtual desenvolvida especificamente para o projeto SUPREME)	Plataforma digital
9	Silverstone et al.^(26)^, 2017, Canadá.	Avaliar os resultados de longo prazo de um programa escolar multimodal para reduzir ideação suicida, depressão e ansiedade entre adolescentes.	Estudo quantitativo, longitudinal, quase-experimental.	Inicialmente, 6.227 alunos Faixa etária: 11 a 18 anos (6º ao 12º ano). 1.884 concluíram todas as quatro avaliações	Escolas públicas	Habilidade para vida Metodologia ativa/multimodal Articulação intersetorial
10	Kahn et al.^(27)^, 2020, Alemanha.	Compreender como o programa Youth Aware of Mental Health, intervenção universal escolar para prevenção do suicídio, interage com diferentes estratégias de enfrentamento	Ensaio clínico randomizado prospectivo	5.654 alunos, entre 14 e 16 anos, de 85 escolas em dez países europeus.	Escolas da Europa	Atividades extracurriculares Habilidade e competência socioemocional
11	Schilling et al.^(28)^, 2021, Chile.	Determinar como as experiências de vida dos adolescentes contribuíram para sua atuação como especialistas por experiência e para o desenvolvimento do Projeto Clã, visando à promoção da saúde mental e à prevenção do suicídio	Estudo de caso qualitativo	Seis alunos, entre 13 e 20 anos	Escolas e ambientes digitais no Chile	Apoio entre pares Plataforma digital Formação de líderes
12	McGillivray et al.^(29)^ 2021, Austrália.	Avaliar o impacto do programa universal Youth Aware of Mental Health de promoção da saúde mental e prevenção do suicídio	Estudo de braço único (*single-arm study*) com pré e pós-teste, tipo de estudo quase-experimental	556 alunos do 9º ano (idades entre 13 e 16 anos), de 18 escolas em quatro regiões de Nova Gales do Sul, Austrália	Escolas de ensino médio.	Habilidade para vida Metodologia ativa/multimodal
13	Kaur^(30)^ 2021, Índia.	Analisar o efeito do treinamento de habilidades para a vida no comportamento suicida de adolescentes.	Quase-experimental (com grupo controle e grupo intervenção – pré e pós-teste)	970 alunos do 9º ano do ensino médio, de 11 escolas	Escolas públicas de ensino médio	Habilidade para vida Rede de apoio
14	Chou et al.^(31)^, 2022, Canadá.	Avaliar a eficácia do programa Core Connectors Initiative	Estudo de métodos mistos	29 alunos, entre 15 e 17 anos	Uma escola pública, uma escola privada e um centro comunitário	Alfabetização em saúde mental Apoio entre pares Rede de apoio
15	Chartier et al.^(32)^, 2022, Canadá.	Analisar, sob a perspectiva de jovens, mentores e anciãos das comunidades indígenas, a abordagem PAX Dream Makers, voltada para promoção da saúde mental e prevenção do suicídio entre jovens.	Estudo qualitativo, do tipo estudo de caso	30 adolescentes (12 a 18 anos) 17 mentores e anciãos adultos de seis comunidades indígenas remotas	Comunidades indígenas das Primeiras Nações em Manitoba, no contexto escolar e comunitário	Habilidade para vida Plataforma digital Formação de líderes
16	Libon et al.^(33)^ 2023, Canadá.	Levantar e documentar perspectivas de jovens sobre a concepção e o desenvolvimento de um modelo de apoio entre pares para promoção da saúde mental e prevenção do suicídio	Estudo qualitativo descritivo, com abordagem de *codesign* participativo, fundamentado na estrutura socioecológica	11 jovens, com idades entre 15 e 24 anos	Comunidades rurais no oeste do Canadá	Apoio entre pares Plataforma digital
17	Walsh et al.^(34)^, 2023, Irlanda.	Explorar as perspectivas dos jovens sobre a promoção da saúde mental escolar e a prevenção do suicídio, utilizando uma abordagem participativa (Photovoice)	Estudo qualitativo, participativo, com análise temática reflexiva e experiencial, fundamentado na abordagem crítica	15 adolescentes de 16 a 19 anos	Ambiente escolar e comunitário na Irlanda	Apoio entre pares
18	Biber e Brandenburg^(35)^, 2024, Estados Unidos.	Descrever o processo de implementação do programa Sources of Strength em uma escola de ensino médio, visando fortalecer fatores de proteção à saúde mental e prevenir comportamento suicida e uso de opioides.	Estudo de caso com avaliação de processo.	1.250 alunos do ensino médio. 135 alunos do ensino médio atuaram como líderes pares (10,8% da escola). 15 mentores universitários, 13 professores e funcionários do ensino médio como mentores adultos	Escola de ensino médio	Articulação intersetorial Treinamento para professores e outros profissionais
19	Bohr et al.^(36)^, 2024. Canadá.	Adaptar culturalmente e avaliar o programa digital SPARX para jovens Inuit, visando fortalecer a saúde mental, a resiliência e a redução do risco de suicídio.	Pesquisa de ação participativa, com métodos mistos.	121 jovens, de 13 a 24 anos. 27 líderes juvenis (15 a 26 anos)	Ambiente escolar, comunitário, incluindo espaços virtuais.	Habilidade para vida Cultura e arte Plataforma digital
20	Knappe et al.^(37)^, 2024, Alemanhã.	Avaliar a eficácia do programa escolar de prevenção ao suicídio HEYLiFE	Estudo quase-experimental, comparativo, intragrupo (pré e pós-intervenção)	218 alunos, de 14 a 18 anos	Escolas públicas	Alfabetização em saúde mental Metodologia ativa/multimodal Habilidade para vida
21	Perrino et al.^(38)^, 2024, Estados Unidos.	Adaptar uma intervenção digital para promover a saúde mental de adolescentes hispânicos, com foco na redução de sintomas de depressão, ansiedade e risco de suicídio.	Estudo de desenvolvimento e adaptação de intervenção.	16 adolescentes (12 a 17 anos) e 16 pais.	Clínicas de atenção primária.	Habilidade para vida Plataforma digital Envolvimento de familiares
22	Wexler et al.^(39)^, 2025, Estados Unidos.	Analisar como a autoeficácia em bem-estar, a autoeficácia na prevenção do suicídio e a participação em comunidades de prática influenciam os comportamentos de prevenção do suicídio e a promoção do bem-estar.	Estudo transversal, quantitativo	398 participantes (entre 15 e 29 anos)	Comunidades indígenas rurais e remotas, em espaços comunitários e redes sociais locais.	Articulação intersetorial Envolvimento de líderes comunitários e familiares

Dados que responderam ao objetivo da revisão foram analisados e apresentados por meio de um resumo narrativo, que descrevia os resultados relacionados com a questão de pesquisa, sendo importante ressaltar que foram apresentados em forma de quadro com base nos dados categorizados para facilitar a compreensão.

### Aspectos Éticos

Esta revisão de escopo obedeceu às diretrizes metodológicas e éticas aplicáveis, respeitando-se as boas práticas em pesquisa científica e os direitos autorais dos estudos incluídos. Por se tratar de uma análise de dados secundários, disponíveis publicamente e sem envolvimento de seres humanos ou informações identificáveis, não houve necessidade de submissão ao Comitê de Ética em Pesquisa, nem de obtenção de Termo de Consentimento Livre e Esclarecido.

## RESULTADOS

A partir da aplicação das estratégias de busca nas bases de dados e nos portais selecionados, foi obtido um total de 3.884 para leitura dos títulos e dos resumos, dos quais 235 estudos foram selecionados para leitura na íntegra, obedecendo aos critérios de elegibilidade e da questão de pesquisa. Desses, foram selecionados 22 estudos para análise e discussão, conforme apresentado no diagrama do fluxo PRISMA-ScR, na Figura 1.

**Figura 1:** Fluxograma PRISMA-ScR do processo de seleção dos estudos incluídos – Florianópolis, SC, Brasil, 2025.

Os estudos surgiram a partir de 1993, com maior ocorrência em 2024 (n = 4; 18%), seguido do ano de 2021 (n = 3; 14%). Com relação à nacionalidade, o Canadá destaca-se (n = 6; 27%); na sequência, os Estados Unidos (n = 5; 23%). Quanto ao tipo de estudo, sete (31,5%) consistiam em estudos quase-experimentais; posteriormente, estudos de casos (n = 3; 14%) e estudos qualitativos participativos (n = 3; 14%). Em relação ao cenário, a escola tem lugar de destaque entre os estudos incluídos (n = 18, 82%). Alguns estudos tiveram mais de um cenário, como comunidades rural, urbana e indígenas, ambiente virtual e universidade, além de outros, conforme consta no Quadro 2.

Sobre a amostra/participantes, observou-se que a maioria dos estudos incluídos (n = 18; 82%) foi desenvolvida com alunos de escolas públicas. Entretanto, alguns também incluíram adolescentes provenientes de comunidades rurais e indígenas, assim como professores, líderes comunitários, adultos jovens e pais. A composição da amostra variou conforme o tipo de estudo e seus objetivos, o que contribuiu para a diversidade das estratégias de promoção da saúde.

Acerca das estratégias de promoção da saúde, emergiram dois enfoques estratégicos, conforme ilustrado na Figura 2, elaborada por meio da ferramenta Google Drawings. As ações identificadas são propostas de forma combinada e articulada, visando ampliar o alcance e potencializar os efeitos das intervenções junto a adolescentes com comportamento suicida. O primeiro enfoque é direcionado aos adolescentes, destacando-se estratégias como desenvolvimento de habilidades para a vida, apoio entre pares, fortalecimento de competências socioemocionais, alfabetização em saúde mental, estímulo ao engajamento e protagonismo juvenil, atividades extracurriculares e práticas artísticas. O segundo enfoque volta-se a diferentes atores sociais envolvidos no cuidado, que incluem campanhas educativas, treinamento para professores e profissionais de diversas áreas, formação de líderes, envolvimento de líderes comunitários e familiares, fortalecimento do apoio social e das redes de suporte, articulação intersetorial, uso de metodologias ativas/multimodais e de plataformas digitais.

**Figura 2:** Estratégias de promoção da saúde direcionadas a adolescentes com comportamento suicida – Florianópolis, SC, Brasil, 2025.

## DISCUSSÃO

Os estudos incluídos nesta revisão de escopo apresentaram estratégias de promoção da saúde para adolescentes com comportamento suicida, com destaque para o cenário escolar, por se tratar de um contexto propício tanto para a implementação dessas estratégias quanto para a prevenção do suicídio^(7,40)^. Na escola, é possível detectar alterações, como problemas de comportamento, queda no desempenho escolar e absenteísmo frequente, que podem ser sinais precoces de sofrimento psíquico em adolescentes^(10)^.

Ao lado do ambiente escolar, o cenário comunitário – que inclui contextos urbanos, rurais e indígenas – ganhou relevo semelhante na implementação de estratégias de promoção da saúde. Nessas comunidades, principalmente quando unidas, o fortalecimento de redes sociais e a participação em atividades coletivas, como práticas religiosas e exercícios em grupo, demonstraram potencial para melhorar o bem-estar físico e emocional e para reduzir o risco de suicídio entre adolescentes^(39,41)^.

Paralelamente, as plataformas digitais e redes sociais emergem como espaços relevantes para conectar adolescentes com seus pares e oferecer suporte emocional^(42)^. Essas ferramentas não só possibilitam a expressão de sentimentos e vivências como favorecem intervenções *on-line*, uma vez que promovem mudanças de atitude e incentivam a busca de ajuda^(43,44)^.

Os serviços de atenção primária sobressaíram-se nos ambientes escolar, comunitário e digital, e surgiram como cenários estratégicos para a promoção da saúde mental de adolescentes com comportamento suicida, por ampliarem o acesso e por possibilitarem a implementação de cuidados^(42)^. Embora ainda existam desafios, iniciativas como modelos colaborativos e soluções inovadoras vêm sendo desenvolvidas^(45)^. No entanto estudo aponta limitações relacionadas à formação e à experiência dos profissionais da atenção primária no manejo de situações como tentativas de suicídio, o que reforça a necessidade de capacitação contínua e fortalecimento das equipes^(46)^.

O mapeamento dos cenários de intervenção evidenciou que escolas, comunidades, ambientes digitais e a atenção primária ampliam o acesso e favorecem a implementação de estratégias de promoção da saúde. Essas estratégias demonstram diversidade e contemplam tanto ações direcionadas aos adolescentes quanto iniciativas que envolvem a participação de outros atores sociais.

Primeiramente, destaca-se o desenvolvimento de habilidades para a vida e competências socioemocionais, que fortalecem a autonomia, a tomada de decisão, o gerenciamento das emoções, a resiliência e a construção de relações interpessoais saudáveis — elementos fundamentais para a promoção da saúde mental e para a redução do risco de suicídio entre adolescentes^(18-21,23,29,30,32,36,38)^. Para potencializar esses resultados, essas habilidades devem ser trabalhadas de forma integrada nos contextos escolar, familiar e nos serviços de saúde, com destaque para o uso dos princípios da terapia cognitivo- comportamental (TCC), reconhecida por sua eficácia no manejo de situações relacionadas ao comportamento suicida e na promoção de fatores de proteção^(10,26,36,43,47,48)^.

Outras estratégias envolvem o fortalecimento dos grupos de apoio entre pares, aliado ao engajamento e ao protagonismo juvenil, os quais favorecem o desenvolvimento psicossocial dos adolescentes ao possibilitar o compartilhamento de experiências difíceis e semelhantes. Além disso, essas iniciativas contribuem para o aprimoramento de habilidades sociais e relacionais e promovem o empoderamento juvenil^(28,34,49)^.

Somadas a essas, outras estratégias mostram-se relevantes, como a alfabetização em saúde mental, atividades extracurriculares e o uso da cultura e da arte, que atuam como ferramentas potentes para o fortalecimento emocional e comunitário dos adolescentes.

A alfabetização em saúde mental constitui uma base essencial para a promoção e contribui para o bem-estar geral dos adolescentes. Intervenções nessa área têm demonstrado aprimorar habilidades como autoeficácia, capacidade de buscar e de compreender informações de saúde, inclusive fortalecendo conexões sociais^(7,37)^. Estratégias criativas, como o uso do teatro, também foram aplicadas para facilitar a alfabetização em saúde mental de forma participativa e contextualizada^(22)^. Outro ponto significativo é que a cultura e a arte aparecem como estratégias promissoras e contribuem positivamente para o bem- estar ao favorecer a expressão de emoções, a valorização cultural e o fortalecimento do orgulho identitário, principalmente em comunidades indígenas^(36)^. Atividades extracurriculares bem estruturadas, como esportes e música, igualmente se destacam como moderadores de humor e promotores de vínculos sociais, já que reforçam fatores protetores contra o sofrimento psíquico^(24)^.

Os estudos têm um enfoque voltado diretamente aos adolescentes, concomitantemente evidenciando a importância de estratégias que ampliem o alcance das ações, como campanhas educativas, programas de capacitação de professores e profissionais de saúde e a formação de líderes comunitários e escolares, fundamentais para sustentar práticas promotoras de saúde de forma integrada e contínua.

Campanhas de prevenção e de conscientização na mídia e em espaços públicos, articuladas em nível governamental, são essenciais para prevenção e promoção, com potencial para influenciar políticas e legislações nas esferas do governo^(47)^. Direcionadas a comunidades rurais, essas ações, aliadas a políticas que ampliem o acesso a atividades estruturadas, fortalecem o engajamento juvenil na promoção da saúde^(21,24)^. Como estratégias universais, alcançam amplos públicos e oferecem informações essenciais para incentivar a busca de ajuda^(21)^.

A capacitação, o treinamento e a formação devem ser estrategicamente direcionados a todos os profissionais e atores sociais, dentro e fora do setor saúde, com o objetivo de aprimorar conhecimentos e competências relacionadas à saúde mental^(47,50)^. Essas estratégias visam, sobretudo, aumentar a conscientização, a autoconfiança e a capacidade dos participantes para identificar riscos de suicídio, intervir precocemente e oferecer suporte adequado^(22,35,44)^. Avaliações de programas de treinamento já implementados demonstram sua eficácia no fortalecimento das práticas de cuidado e na ampliação da resposta comunitária a situações de crise^(31,43)^.

Além das campanhas, destaca-se a importância da capacitação e do envolvimento de líderes comunitários e familiares, com foco em fortalecer a confiança dos adolescentes em seus cuidadores e em reparar rupturas nos vínculos afetivos, fatores associados ao risco de ideação e a tentativas de suicídio^(43,44)^. Estratégias que estimulam o engajamento familiar e comunitário, bem como o discurso aberto sobre o suicídio, contribuem para a criação de ambientes sociais acolhedores e favoráveis à promoção da saúde mental^(7)^. Esses espaços, ao possibilitarem a observação precoce de sinais de sofrimento, tornam-se essenciais na prevenção.

A articulação interdisciplinar e intersetorial entre saúde, educação, assistência social e organizações comunitárias potencializa as ações de promoção da saúde e deve ser incentivada. Essa integração amplia a participação coletiva no planejamento e no desenvolvimento de estratégias, de modo que se fortalecem as redes de apoio e a efetividade das intervenções^(23,47)^. A abordagem intersetorial, territorial e interdisciplinar contribui para o fortalecimento de fatores de proteção e, consequentemente, para a redução das taxas de suicídio entre adolescentes, especialmente quando há colaboração ativa entre os setores e a comunidade^(26,39,51)^.

As metodologias ativas, como estudo de caso, dramatizações e aprendizagem baseada em problemas, têm demonstrado resultados promissores na capacitação de adolescentes para a discussão e a reflexão sobre o suicídio, mas ainda necessitam de maior implementação e integração aos planos de ensino das disciplinas escolares^(10,22,29,37)^. De forma complementar, estratégias inovadoras como plataformas digitais, redes sociais e aplicativos voltados a adolescentes e familiares têm-se destacado por facilitarem o aprendizado de habilidades, ampliarem redes de apoio e oferecerem serviços *on-line* 24 horas, configurando-se como recursos promissores para promoção da saúde mental^(25,28,33,38)^. No entanto, essas iniciativas enfrentam desafios, como questões de acessibilidade, conectividade, segurança dos dados, necessidade de investimento financeiro e adequações culturais para garantir efetividade em diferentes contextos^(36,52)^.

Embora existam marcos normativos sólidos para a implementação de políticas e estratégias de promoção da saúde no Brasil, a articulação institucional, a disponibilização de recursos, a capacidade técnica e a coordenação intersetorial ainda representam desafios significativos para sua efetivação no âmbito do Sistema Único de Saúde (SUS)^(53)^. De modo semelhante, estudo sobre promoção da saúde mental indica que a implementação de estratégias eficazes esbarra em limitações de recursos, na escassez de evidências de custo-efetividade e em barreiras contextuais que dificultam a adaptação de modelos internacionais às realidades locais^(54)^.

As estratégias identificadas nesta revisão reforçam o papel da enfermagem na promoção da saúde mental de adolescentes com comportamento suicida. Atuando em diferentes cenários, o enfermeiro contribui para ampliar o acesso ao cuidado, para fortalecer vínculos e para articular redes de apoio. Essas ações exigem escuta sensível, formação contínua e atuação intersetorial, o que reafirma o compromisso ético e político da profissão com a vida e com o bem-estar dos sujeitos em sofrimento.

Contudo, para o fortalecimento e a ampliação dessas práticas, é necessário reconhecer as lacunas ainda existentes na produção científica sobre o tema. Embora esta revisão de escopo tenha mapeado diversas estratégias de promoção da saúde para adolescentes com comportamento suicida, é importante reconhecer as limitações. Destaca-se como limitação relevante a não recuperação de parte dos estudos identificados, sobretudo os mais antigos. Ao todo, 38 documentos, majoritariamente das décadas de 1980 e 1990, não puderam ser acessados devido à indisponibilidade do texto completo nas bases de dados e nos repositórios institucionais. Apesar da realização de buscas complementares, não foi possível obtê-los, o que pode ter reduzido a amplitude histórica do mapeamento.

Ademais, a predominância de estudos conduzidos em países de alta renda limita a aplicação direta das estratégias ao contexto brasileiro, marcado por especificidades epidemiológicas, socioculturais e estruturais. Tal cenário impõe desafios para o desenvolvimento de políticas e de práticas de promoção da saúde voltadas aos adolescentes com comportamento suicida e reforça a necessidade de pesquisas nacionais e regionais que considerem desigualdades socioeconômicas, determinantes sociais da saúde e a organização dos sistemas públicos, como o SUS. Investigações futuras podem contribuir para a adaptação, a validação e a contextualização de estratégias internacionais de sorte que se tornem mais sensíveis às realidades locais e culturalmente adequadas.

## CONCLUSÃO

Os estudos analisados nesta revisão apresentam dois enfoques estratégicos que se combinam e se articulam para a prevenção e para a promoção da saúde. O primeiro, relacionado diretamente ao adolescente, visa ao fortalecimento de recursos internos e relacionais por meio do desenvolvimento de habilidades para a vida, apoio entre pares, competências socioemocionais, alfabetização em saúde mental, estímulo ao engajamento e ao protagonismo juvenil, além de atividades extracurriculares e de práticas artísticas. Essas estratégias contribuem para o empoderamento dos adolescentes e para o fortalecimento de sua resiliência diante do sofrimento psíquico. O segundo enfoque é direcionado a diferentes atores sociais que compõem o contexto ampliado de cuidado, como professores, profissionais da saúde e educação, líderes comunitários e família, que potencializam a criação de ambientes de cuidado e a mobilização comunitária, de forma que se amplie o alcance e a eficácia das ações de promoção da saúde, por meio de campanhas educativas, de treinamento para professores e profissionais de diversas áreas, de formação de líderes, de apoio social e das redes de suporte, de articulação intersetorial, de uso de metodologias ativas/ multimodais e de plataformas digitais.

Acrescenta-se que, embora o ambiente escolar tenha sido amplamente explorado como espaço de intervenção, destaca-se a necessidade de ampliar as estratégias de promoção da saúde para outros contextos, como os serviços especializados em saúde mental, aos quais muitos adolescentes chegam em situação crítica de sofrimento psíquico e com risco de iminente suicídio.

Destaca-se, ainda, a necessidade de estratégias que ultrapassem a capacitação de profissionais de saúde por meio da incorporação ativa das famílias no processo de cuidado. Apesar do reconhecimento de seu papel essencial, observa-se uma carência de ações sistemáticas que as incluam como protagonistas na promoção da saúde de adolescentes com comportamento suicida. Essa lacuna evidencia a urgência de desenvolver abordagens integradas, intersetoriais e culturalmente sensíveis, capazes de impulsionar respostas às demandas complexas desse público e de contribuir para a redução das situações de suicídio entre adolescentes.

## DISPONIBILIDADE DE DADOS

Os dados que sustentam os resultados deste estudo estão disponíveis de forma aberta no Open Science Framework (OSF), em: https://doi.org/10.17605/OSF.IO/J9MQB.

# Translation: Estrategias de promoción de la salud para adolescentes con comportamiento suicida: revisión de alcance

**DOI:** 10.1590/1980-220X-REEUSP-2025-0338es

**Authors:** Erika Augusta do Amaral Coelho Bezerra, Ivonete Teresinha Schulter Buss Heidemann, Aldalice Aguiar de Souza, Eliana Rosa da Fonseca, Camila Trindade Coelho, Paola Margarita Oñate Daza, Richard Augusto Thomann Beckert, Alaidistânia Aparecida Ferreira

**Affiliations:** 1 Universidade Federal de Santa Catarina, Florianópolis, SC, Brasil.; 2 Universidade do Estado do Amazonas, Manaus, AM, Brasil.; 3 Universidade Federal do Rio de Janeiro, Rio de Janeiro, RJ, Brasil.; 4 Universidade Federal do Amazonas, Manaus, AM, Brasil.

**Keywords:** Adolescente, Estrategias de Salud, Prevención del Suicidio, Promoción de la Salud, Suicidio

## Abstract

**Objetivo:**

Mapear estrategias para promover la salud de los adolescentes con conductas suicidas.

**Método:**

Revisión del alcance de acuerdo con las directrices metodológicas de JBI, sin limitaciones de idioma ni restricciones de tiempo. Criterios de inclusión para los estudios: adolescentes de entre diez y 19 años con conducta suicida para explorar el concepto de promoción de la salud, en cualquier servicio de salud, pero sin limitar este contexto. Fuentes consultadas: MEDLINE/PubMed, PubMed Central/NLM, CINAHL, Academic Search Premier, Academic Source/EBSCO, Scopus, Embase/Elsevier, LILACS, BDENF, INDEXPSI, CUMED, BINACIS, LIPECS, MedCarib, BBO, ARGMSAL, BDNPAR, BIGG, BRISA, HomeoIndex, PAHOIRIS, SES-SPBVS, SciELO, PsycInfo/APA.

**Resultados:**

De los 22 artículos seleccionados, surgieron dos enfoques estratégicos dirigidos a los adolescentes: el desarrollo de habilidades para la vida (apoyo entre pares, alfabetización en salud mental, participación juvenil) y la implicación de diferentes actores sociales (familia, profesionales, líderes comunitarios, redes de apoyo intersectoriales).

**Conclusión:**

Se identificaron y mapearon estrategias de promoción de la salud utilizando dos enfoques estratégicos: primero, se dirigieron a los adolescentes; luego, involucraron a diferentes actores sociales, ampliando el alcance de la promoción de la salud.

## INTRODUCCIÓN

Las estrategias de promoción de la salud cobran importancia en la Atención Primaria de Salud (APS) debido a su fuerte convergencia con sus principios, que favorecen el acceso a los servicios y la implementación de acciones integradas^(1)^. La APS también permite articular intervenciones orientadas tanto a cuestiones individuales como colectivas, con el objetivo de mejorar las condiciones de vida y fortalecer la salud comunitaria^(2,3)^. En este sentido, la promoción de la salud (PS) amplía el concepto de salud y se configura como una estrategia prometedora para enfrentar los desafíos de la salud pública al analizar los determinantes de la salud y potenciar las acciones intersectoriales^(4,5)^.

Con el propósito de mejorar las condiciones de vida y de salud en las dimensiones individuales y colectivas, la PS establece una gama de estrategias políticas con directrices y ejes transversales. De este modo, las estrategias se caracterizan por la cooperación entre diferentes sectores y dentro del propio sector de la salud, por la articulación con redes de protección social y por el fomento de una amplia participación y del control social. Su enfoque principal es reducir las vulnerabilidades y los riesgos derivados de determinantes multifacéticos: sociales, económicos, políticos, culturales y ambientales.

Además, prioriza el empoderamiento de la persona y la comunidad para que tengan un mayor control sobre su proceso de vida. Partiendo de una comprensión integral del proceso de salud-enfermedad y de sus determinantes, la SP busca integrar y valorizar el conocimiento popular junto con el conocimiento técnico y científico mediante acciones que abarcan el cambio de comportamiento, las intervenciones colectivas y la implementación de políticas públicas integradas^(3,4)^.

En la APS, especialmente en el ámbito de la salud mental, la necesidad de atención desde la perspectiva de la PS, orientada por el paradigma psicosocial, hace que las personas con sufrimiento psíquico se conviertan en sujetos activos, integrados en sus territorios, lo que permite nuevas formas de expresión de la vida, la autonomía y la socialización^(6)^.

Las personas con sufrimiento psíquico, principalmente los niños y adolescentes, requieren estrategias amplias e intersectoriales orientadas al desarrollo de habilidades y competencias sociales, al fortalecimiento, a la superación de dificultades y al apoyo social, con un enfoque en la PS^(4,7)^, tomando en cuenta su singularidad y el territorio en el que se encuentran insertadas.

Entre las diversas manifestaciones de sufrimiento psíquico, sobre todo entre niños y adolescentes, el suicidio se destaca como una de sus formas más graves, dada su complejidad y su impacto social, debido a la inmadurez neuropsicosocial y emocional de este grupo. De 2011 a 2022, se observó un aumento en las tasas de suicidio en todas las regiones de Brasil, con mayor incidencia entre los jóvenes de 10 a 24 años, lo que significó un incremento del 6,14 %^(8)^. En la región de las Américas, entre 2000 y 2019, se observó un aumento del 17 % en los casos, mientras que, en Brasil, hubo un crecimiento significativo del 43 % en el número de suicidios^(9)^ durante ese mismo período.

El suicidio entre adolescentes refleja múltiples factores que contribuyen a él, como los trastornos mentales, los conflictos familiares, la violencia, el *acoso (bullying)*, la exclusión social, el consumo de sustancias como el alcohol y las drogas, además del impacto de las tecnologías a través de los medios de comunicación y las redes sociales, que con frecuencia se asocian con efectos negativos sobre la salud mental^(10,11)^. Ante este panorama, se hace evidente la necesidad de identificar estrategias que no se limiten únicamente a la prevención de los problemas de salud, sino que también se centren en la salud pública, que sean integrales y estén coordinadas con diferentes sectores capaces de abordar la complejidad de estos problemas de salud pública^(10,12)^.

Se realizaron búsquedas preliminares con el objetivo de identificar revisiones publicadas o protocolos en curso sobre el mismo tema, para garantizar la originalidad del estudio y evitar la duplicación de esfuerzos. Esta búsqueda incluyó las bases de datos MEDLINE/PubMed, la Base de Datos Cochrane de Revisiones Sistemáticas, el Portal Regional de la Biblioteca Virtual en Salud (BVS), PROSPERO y JBI Evidence Synthesis, con lo cual se identificaron dos protocolos de revisión: uno enfocado en estrategias intersectoriales de atención a adolescentes con comportamiento suicida y autolesivo, y otro en intervenciones destinadas a la prevención y el manejo del suicidio y la automutilación en contextos educativos.

Aunque están relacionados con el tema, estos estudios no abordan el enfoque específico de esta propuesta. Por lo tanto, no se encontraron revisiones actuales o en curso que aborden estrategias de promoción de la salud dirigidas a adolescentes con comportamiento suicida, lo que pone de manifiesto una brecha en el conocimiento y refuerza la relevancia de esta investigación. Por lo tanto, este estudio tiene como objetivo mapear estrategias de promoción de la salud dirigidas a adolescentes con comportamiento suicida.

## MÉTODO

### Tipo de Estudio

Se trata de una revisión de alcance realizada de acuerdo con las directrices metodológicas del JBI^(13)^, con el fin de garantizar el rigor metodológico, la claridad y la organización del proceso de revisión, y reportada conforme a las orientaciones de la *lista de verificación* PRISMA Extension for Scoping Reviews (PRISMA-ScR)^(14)^. El protocolo de esta revisión se registró en la plataforma Open Science Framework (OSF) con el DOI https://doi.org/10.17605/OSF.IO/J9MQB.

### Pregunta de Investigación

La pregunta de investigación se definió de acuerdo con el acrónimo PCC (población/concepto/contexto), en el que la población (P) son «adolescentes con comportamiento suicida»; el concepto (C) alude a la «promoción de la salud»; y el contexto (C) se refiere a cualquier servicio de salud mental en todos los niveles de atención, sin limitarse a ellos. Se estableció la siguiente pregunta de investigación: ¿Cuáles son las estrategias de promoción de la salud para adolescentes con comportamiento suicida?

### Criterios de Elegibilidad

Población: esta revisión considera estudios que incluyen a adolescentes de 10 a 19 años^(15)^ con comportamiento suicida. La Organización Mundial de la Salud (OMS)^(16)^ destaca el suicidio como un grave problema de salud pública que requiere una respuesta coordinada. Con intervenciones oportunas, basadas en la evidencia y de bajo costo, es posible reducir significativamente la incidencia de suicidios. Aunque muchos incidentes son impulsivos, el comportamiento suicida está fuertemente asociado a desastres, conflictos, violencia, abuso o pérdida y a una sensación de aislamiento. La OMS establece intervenciones eficaces basadas en la evidencia, lo que incluye la identificación, la evaluación, el manejo y el seguimiento temprano de cualquier persona afectada por comportamientos suicidas^(16)^.

Concepto – Se incluyeron estudios que abordaran estrategias de promoción de la salud para adolescentes con comportamiento suicida. Se tomó en cuenta la definición de la Carta de Ottawa, que define la promoción de la salud como el «proceso de empoderamiento de las comunidades para mejorar su calidad de vida y su salud, incluida la participación en el control de dicho proceso»^(4,17)^.

Contexto: Los estudios incluidos se realizaron en cualquier lugar que ofreciera servicios de salud mental en cualquier nivel de atención. Se consideraron igualmente los servicios en cualquier nivel de atención médica, teniendo en cuenta el rango de edad de los participantes, pero sin limitarse a ellos.

Se consideraron estudios con diseños experimentales y cuasi-experimentales (ensayos clínicos aleatorizados, ensayos controlados no aleatorizados, estudios de antes y después y series temporales interrumpidas), observacionales analíticos (cohortes prospectivas y retrospectivas, estudios de casos y controles y estudios transversales analíticos) y observacionales descriptivos (series de casos, informes de casos y estudios transversales descriptivos). Se incluyeron estudios cualitativos (fenomenología, teoría fundamentada, etnografía, descripción cualitativa e investigación-acción), además de textos teóricos, artículos de opinión, disertaciones y tesis. Se excluyeron las revisiones sistemáticas, de alcance e integrativas, así como los resúmenes de eventos, cartas y editoriales.

### Estrategia de Búsqueda

La estrategia de búsqueda se llevó a cabo en tres etapas, con el objetivo de localizar estudios publicados y no publicados, en colaboración con una bibliotecaria especializada, certificada por el JBI en métodos de revisión. Inicialmente, se realizó una búsqueda preliminar en los portales: MEDLINE/ PubMed, Cochrane Database of Systematic Reviews, Portal Regional de la Biblioteca Virtual en Salud (BVS), PROSPERO y JBI Evidence Synthesis. En la segunda etapa, la estrategia definitiva presentada en el Cuadro 1 se adaptó a las bases de datos y portales: MEDLINE/PubMed, PubMed Central/NLM, CINAHL, Academic Search Premier, Fonte Acadêmica/EBSCO, Scopus, Embase/Elsevier, LILACS, BDENF, INDEXPSI, CUMED, BINACIS, LIPECS, MedCarib, BBO, ARGMSAL, BDNPAR, BIGG, BRISA, HomeoIndex, PAHOIRIS, SES-SPLILACS, BDENF, Index Psicologia/ BVS, SciELO y PsycInfo/APA. La literatura gris se exploró mediante la plataforma Science.gov; además, se incorporó la herramienta de búsqueda Google Scholar. No hubo restricciones de idioma ni de período de publicación. Por último, en la tercera etapa, se realizó una búsqueda manual en las referencias de los estudios incluidos. La búsqueda se llevó a cabo el 23 de enero de 2025.

**Cuadro 1:** Estrategias de búsqueda para bases de datos y portales – Florianópolis, SC, Brasil, 2025.

Bases de datos/Portales	Estrategias	Total
BVS	((adolescente OR adolescência OR adolescente* OR jovem OR jovens OR juventude OR joven OR jóvenes OR juventud OR jeunes OR jeunesse OR adolescent OR adolescence OR adolescent* OR teen* OR teenager* OR youth*) AND (“Ideação Suicida” OR “Ideación Suicida” OR “Ideas Suicidas” OR “Idéation suicidaire” OR “Idéation de suicide” OR “Idées suicidaires” OR “Comportamento Autodestrutivo” OR “Autoagressão Intencional” OR autolesão OR “Autolesão Intencional” OR “Autolesão não Suicida” OR “Conduta Autolesiva” OR “Ferimento Autoinfligido não Suicida” OR “Lesão Autoinfligida não Suicida” OR “Conducta Autodestructiva” OR “Autolesión Intencional” OR “Autolesión no Suicida” OR autolesiones OR “Herimiento Autoinferido no Suicida” OR “Herimientos Autoinferidos” OR “Comportement auto-agressif” OR “Automutilation délibérée” OR “Automutilation non suicidaire” OR “Comportement autoagressif” OR “Comportement autodestructeur” OR “Comportement d’auto-agression” OR “Comportement d’autoagression” OR “Comportement d’automutilation” OR suicídio OR suicídio* OR suicide OR “Suicidal Ideation” OR “Suicidal Ideations” OR “Self-Injurious Behavior” OR “Deliberate Self Harm” OR “Deliberate Self-Harm” OR “Intentional Self Harm” OR “Intentional Self Injuries” OR “Intentional Self Injury” OR “Non Suicidal Self Injury” OR “Non-Suicidal Self Injuries” OR “Non-Suicidal Self Injury” OR “Nonsuicidal Self Injuries” OR “Nonsuicidal Self Injury” OR “Self Destructive Behavior” OR “Self Harm” OR “Self Injurious Behavior” OR “Self Injury” OR “Self-Destructive Behavior” OR “Self-Destructive Behaviors” OR self-injuries OR “Self-Injurious Behaviors” OR self-injury OR suicide OR suicide* OR “Prevenção do Suicídio” OR “Conscientização sobre Suicídio” OR “Prevenção contra Suicídio” OR “Prevención del Suicidio” OR “Conciencia Sobre el Suicidio” OR “Prévention du suicide” OR “Suicide Prevention” OR “Suicide Awareness” OR “Suicide Preventions”)) AND ((“Promoção da Saúde” OR “Ambientes Apoiadores de Saúde” OR “Ambientes de Apoio à Saúde” OR “Campanhas de Saúde” OR “Item Promocional” OR “Itens Promocionais” OR “Programas de Bem-Estar” OR “Promoção do Bem Estar” OR “Promoção em Saúde” OR “Promoción de la Salud” OR “Ambientes de Apoyo a la Salud” OR “Campanas de Salud” OR “Entornos Apoyadores de la Salud” OR “Entornos de Apoyo a la Salud” OR “Items Promocionales” OR “Programas de Bienestar” OR “Promoción del Bienestar” OR “Promotion de la santé” OR “Campagnes de santé” OR “Campagnes pour la santé” OR “Points promotionnels” OR “Programmes de bien-être” OR “pessoas saudáveis” OR “programas para pessoas saudáveis” OR “Éducation Environnementale” OR “Educação em Saúde” OR “Educar para a Saúde” OR “Educação para a Saúde” OR “Educação Sanitária” OR “Educación en Salud” OR “Educación para la Salud” OR “Educación para la Salud Comunitaria” OR “Educación Sanitaria” OR “Éducation pour la santé” OR “Éducation communautaire en santé” OR “Éducation en santé” OR “Éducation pour la santé au sein de la communauté” OR “Éducation sanitaire” OR “Éducation sanitaire communautaire” OR “Formation communautaire en santé” OR “Health Promotion” OR “Health Campaign” OR “Health Campaigns” OR “Health Promotions” OR “Health-Supportive Environments” OR “Promotion of Health” OR “Promotional Item” OR “Promotional Items” OR “Wellness Program” OR “Wellness Programs” OR “healthy people 2010”or “healthy people programmes” OR “healthy people programs” OR “Health Education” OR “Community Health Education”)) AND db:(“LILACS” OR “BDENF” OR “INDEXPSI” OR “CUMED” OR “BINACIS” OR “LIPECS” OR “MedCarib” OR “BBO” OR “ARGMSAL” OR “BDNPAR” OR “BIGG” OR “BRISA” OR “HomeoIndex” OR “PAHOIRIS” OR “SES-SP”) AND instance:”lilacsplus”	714
Academic Searche Premier ( ) CINAHL with Full Text Fonte Acadêmica ( )	((TI Adolescent OR Adolescence OR Adolescent* OR Teen* OR Teenager* OR Youth* OR SU Adolescent OR Adolescence OR Adolescent* OR Teen* OR Teenager* OR Youth* OR AB Adolescent OR Adolescence OR Adolescent* OR Teen* OR Teenager* OR Youth*) AND (TI “Suicidal Ideation” OR “Suicidal Ideations” OR “Self-Injurious Behavior” OR “Deliberate Self Harm” OR “Deliberate Self-Harm” OR “Intentional Self Harm” OR “Intentional Self Injuries” OR “Intentional Self Injury” OR “Non Suicidal Self Injury” OR “Non-Suicidal Self Injuries” OR “Non-Suicidal Self Injury” OR “Nonsuicidal Self Injuries” OR “Nonsuicidal Self Injury” OR “Self Destructive Behavior” OR “Self Harm” OR “Self Injurious Behavior” OR “Self Injury” OR “Self-Destructive Behavior” OR “Self-Destructive Behaviors” OR Self-Injuries OR “Self-Injurious Behaviors” OR Self-Injury OR Suicide OR Suicide* OR “Suicide Prevention” OR “Awareness, Suicide” OR “Prevention, Suicide” OR “Suicide Awareness” OR “Suicide Preventions” OR SU “Suicidal Ideation” OR “Suicidal Ideations” OR “Self-Injurious Behavior” OR “Deliberate Self Harm” OR “Deliberate Self-Harm” OR “Intentional Self Harm” OR “Intentional Self Injuries” OR “Intentional Self Injury” OR “Non Suicidal Self Injury” OR “Non-Suicidal Self Injuries” OR “Non-Suicidal Self Injury” OR “Nonsuicidal Self Injuries” OR “Nonsuicidal Self Injury” OR “Self Destructive Behavior” OR “Self Harm” OR “Self Injurious Behavior” OR “Self Injury” OR “Self-Destructive Behavior” OR “Self-Destructive Behaviors” OR Self-Injuries OR “Self-Injurious Behaviors” OR Self-Injury OR Suicide OR Suicide* OR “Suicide Prevention” OR “Awareness, Suicide” OR “Prevention, Suicide” OR “Suicide Awareness” OR “Suicide Preventions” OR AB “Suicidal Ideation” OR “Suicidal Ideations” OR “Self-Injurious Behavior” OR “Deliberate Self Harm” OR “Deliberate Self-Harm” OR “Intentional Self Harm” OR “Intentional Self Injuries” OR “Intentional Self Injury” OR “Non Suicidal Self Injury” OR “Non-Suicidal Self Injuries” OR “Non-Suicidal Self Injury” OR “Nonsuicidal Self Injuries” OR “Nonsuicidal Self Injury” OR “Self Destructive Behavior” OR “Self Harm” OR “Self Injurious Behavior” OR “Self Injury” OR “Self-Destructive Behavior” OR “Self-Destructive Behaviors” OR Self-Injuries OR “Self-Injurious Behaviors” OR Self-Injury OR Suicide OR Suicide* OR “Suicide Prevention” OR “Awareness, Suicide” OR “Prevention, Suicide” OR “Suicide Awareness” OR “Suicide Preventions”)) AND (TI “Health Promotion” OR “Health Campaign” OR “Health Campaigns” OR “Health Promotions” OR “Health-Supportive Environments” OR “Promotion of Health” OR “Promotional Item” OR “Promotional Items” OR “Wellness Program” OR “Wellness Programs” OR “healthy people 2010”OR “healthy people programmes” OR “healthy people programs” OR “Health Education” OR “Community Health Education” OR SU “Health Promotion” OR “Health Campaign” OR “Health Campaigns” OR “Health Promotions” OR “Health-Supportive Environments” OR “Promotion of Health” OR “Promotional Item” OR “Promotional Items” OR “Wellness Program” OR “Wellness Programs” OR “healthy people 2010”OR “healthy people programmes” OR “healthy people programs” OR “Health Education” OR “Community Health Education” OR AB “Health Promotion” OR “Health Campaign” OR “Health Campaigns” OR “Health Promotions” OR “Health-Supportive Environments” OR “Promotion of Health” OR “Promotional Item” OR “Promotional Items” OR “Wellness Program” OR “Wellness Programs” OR “healthy people 2010”OR “healthy people programmes” OR “healthy people programs” OR “Health Education” OR “Community Health Education”)	1099
EMBASE	(‘adolescent’/exp OR adolescent OR ‘adolescence’/exp OR adolescence OR adolescent* OR teen* OR teenager* OR youth*) AND (‘suicidal ideation’/exp OR ‘suicidal ideation’ OR ‘suicidal ideations’ OR ‘self-injurious behavior’/exp OR ‘self-injurious behavior’ OR ‘deliberate self harm’/exp OR ‘deliberate self harm’ OR ‘deliberate self-harm’/exp OR ‘deliberate self-harm’ OR ‘intentional self harm’ OR ‘intentional self injuries’ OR ‘intentional self injury’ OR ‘non suicidal self injury’/exp OR ‘non suicidal self injury’ OR ‘non-suicidal self injuries’ OR ‘non-suicidal self injury’/exp OR ‘non-suicidal self injury’ OR ‘nonsuicidal self injuries’ OR ‘nonsuicidal self injury’/exp OR ‘nonsuicidal self injury’ OR ‘self destructive behavior’/exp OR ‘self destructive behavior’ OR ‘self harm’/exp OR ‘self harm’ OR ‘self injurious behavior’/exp OR ‘self injurious behavior’ OR ‘self-destructive behavior’ OR ‘self-destructive behaviors’ OR ‘self injuries’ OR ‘self-injurious behaviors’ OR ‘self injury’/exp OR ‘self injury’ OR ‘suicide’/exp OR suicide OR suicide* OR ‘suicide prevention’/exp OR ‘suicide prevention’ OR ‘awareness, suicide’ OR ‘prevention, suicide’ OR ‘suicide awareness’ OR ‘suicide preventions’) AND (‘health promotion’/exp OR ‘health promotion’ OR ‘health campaign’ OR ‘health campaigns’ OR ‘health promotions’ OR ‘health-supportive environments’ OR ‘promotion of health’ OR ‘promotional item’ OR ‘promotional items’/exp OR ‘promotional items’ OR ‘wellness program’ OR ‘wellness programs’ OR ‘healthy people 2010’/exp OR ‘healthy people 2010’ OR ‘healthy people programmes’/exp OR ‘healthy people programmes’ OR ‘healthy people programs’/exp OR ‘healthy people programs’ OR ‘health education’/exp OR ‘health education’ OR ‘community health education’) AND ([article]/lim OR [article in press]/lim OR [conference paper]/lim OR [conference review]/lim OR [review]/lim OR [preprint]/lim)	1825
Science	Title: (adolescent OR adolescence OR adolescent* OR teen* OR teenager* OR youth*) AND (“Suicidal Ideation” OR “Suicidal Ideations” OR “Self-Injurious Behavior” OR “Deliberate Self Harm” OR “Deliberate Self-Harm” OR “Intentional Self Harm” OR “Intentional Self Injuries” OR “Intentional Self Injury” OR “Non Suicidal Self Injury” OR “Non-Suicidal Self Injuries” OR “Non-Suicidal Self Injury” OR “Nonsuicidal Self Injuries” OR “Nonsuicidal Self Injury” OR “Self Destructive Behavior” OR “Self Harm” OR “Self Injurious Behavior” OR “Self Injury” OR “Self-Destructive Behavior” OR “Self-Destructive Behaviors” OR self-injuries OR “Self-Injurious Behaviors” OR self-injury OR suicide OR suicide* OR “Suicide Prevention” OR “Awareness, Suicide” OR “Prevention, Suicide” OR “Suicide Awareness” OR “Suicide Preventions”) AND (“Health Promotion” OR “Health Campaign” OR “Health Campaigns” OR “Health Promotions” OR “Health-Supportive Environments” OR “Promotion of Health” OR “Promotional Item” OR “Promotional Items” OR “Wellness Program” OR “Wellness Programs” OR “healthy people 2010” OR “healthy people programmes” OR “healthy people programs” OR “Health Education” OR “Community Health Education”)	270
PSYCINFO	(Title: adolescent OR Title: adolescence OR Title: adolescent* OR Title: teen* OR Title: teenager* OR Title: youth*) AND (Title: “Suicidal Ideation” OR Title: “Suicidal Ideations” OR Title: “Self-Injurious Behavior” OR Title: “Deliberate Self Harm” OR Title: “Deliberate Self-Harm” OR Title: “Intentional Self Harm” OR Title: “Intentional Self Injuries” OR Title: “Intentional Self Injury” OR Title: “Non Suicidal Self Injury” OR Title: “Non-Suicidal Self Injuries” OR Title: “Non-Suicidal Self Injury” OR Title: “Nonsuicidal Self Injuries” OR Title: “Nonsuicidal Self Injury” OR Title: “Self Destructive Behavior” OR Title: “Self Harm” OR Title: “Self Injurious Behavior” OR Title: “Self Injury” OR Title: “Self-Destructive Behavior” OR Title: “Self-Destructive Behaviors” OR Title: self-injuries OR Title: “Self-Injurious Behaviors” OR Title: self-injury OR Title: suicide OR Title: suicide* OR Title: “Suicide Prevention” OR Title: “Awareness, Suicide” OR Title: “Prevention, Suicide” OR Title: “Suicide Awareness” OR Title: “Suicide Preventions”) AND (Title: “Health Promotion” OR Title: “Health Campaign” OR Title: “Health Campaigns” OR Title: “Health Promotions” OR Title: “Health-Supportive Environments” OR Title: “Promotion of Health” OR Title: “Promotional Item” OR Title: “Promotional Items” OR Title: “Wellness Program” OR Title: “Wellness Programs” OR Title: “healthy people 2010” OR Title: “healthy people programmes” OR Title: “healthy people programs” OR Title: “Health Education” OR Title: “Community Health Education”) OR (Abstract: adolescent OR Abstract: adolescence OR Abstract: adolescent* OR Abstract: teen* OR Abstract: teenager* OR Abstract: youth*) AND (Abstract: “Suicidal Ideation” OR Abstract: “Suicidal Ideations” OR Abstract: “Self-Injurious Behavior” OR Abstract: “Deliberate Self Harm” OR Abstract: “Deliberate Self-Harm” OR Abstract: “Intentional Self Harm” OR Abstract: “Intentional Self Injuries” OR Abstract: “Intentional Self Injury” OR Abstract: “Non Suicidal Self Injury” OR Abstract: “Non-Suicidal Self Injuries” OR Abstract: “Non-Suicidal Self Injury” OR Abstract: “Nonsuicidal Self Injuries” OR Abstract: “Nonsuicidal Self Injury” OR Abstract: “Self Destructive Behavior” OR Abstract: “Self Harm” OR Abstract: “Self Injurious Behavior” OR Abstract: “Self Injury” OR Abstract: “Self-Destructive Behavior” OR Abstract: “Self-Destructive Behaviors” OR Abstract: self-injuries OR Abstract: “Self-Injurious Behaviors” OR Abstract: self-injury OR Abstract: suicide OR Abstract: suicide* OR Abstract: “Suicide Prevention” OR Abstract: “Awareness, Suicide” OR Abstract: “Prevention, Suicide” OR Abstract: “Suicide Awareness” OR Abstract: “Suicide Preventions”) AND (Abstract: “Health Promotion” OR Abstract: “Health Campaign” OR Abstract: “Health Campaigns” OR Abstract: “Health Promotions” OR Abstract: “Health-Supportive Environments” OR Abstract: “Promotion of Health” OR Abstract: “Promotional Item” OR Abstract: “Promotional Items” OR Abstract: “Wellness Program” OR Abstract: “Wellness Programs” OR Abstract: “healthy people 2010” OR Abstract: “healthy people programmes” OR Abstract: “healthy people programs” OR Abstract: “Health Education” OR Abstract: “Community Health Education”) OR (Keywords: adolescent OR Keywords: adolescence OR Keywords: adolescent* OR Keywords: teen* OR Keywords: teenager* OR Keywords: youth*) AND (Keywords: “Suicidal Ideation” OR Keywords: “Suicidal Ideations” OR Keywords: “Self-Injurious Behavior” OR Keywords: “Deliberate Self Harm” OR Keywords: “Deliberate Self-Harm” OR Keywords: “Intentional Self Harm” OR Keywords: “Intentional Self Injuries” OR Keywords: “Intentional Self Injury” OR Keywords: “Non Suicidal Self Injury” OR Keywords: “Non-Suicidal Self Injuries” OR Keywords: “Non-Suicidal Self Injury” OR Keywords: “Nonsuicidal Self Injuries” OR Keywords: “Nonsuicidal Self Injury” OR Keywords: “Self Destructive Behavior” OR Keywords: “Self Harm” OR Keywords: “Self Injurious Behavior” OR Keywords: “Self Injury” OR Keywords: “Self-Destructive Behavior” OR Keywords: “Self-Destructive Behaviors” OR Keywords: self-injuries OR Keywords: “Self-Injurious Behaviors” OR Keywords: self-injury OR Keywords: suicide OR Keywords: suicide* OR Keywords: “Suicide Prevention” OR Keywords: “Awareness, Suicide” OR Keywords: “Prevention, Suicide” OR Keywords: “Suicide Awareness” OR Keywords: “Suicide Preventions”) AND (Keywords: “Health Promotion” OR Keywords: “Health Campaign” OR Keywords: “Health Campaigns” OR Keywords: “Health Promotions” OR Keywords: “Health-Supportive Environments” OR Keywords: “Promotion of Health” OR Keywords: “Promotional Item” OR Keywords: “Promotional Items” OR Keywords: “Wellness Program” OR Keywords: “Wellness Programs” OR Keywords: “healthy people 2010” OR Keywords: “healthy people programmes” OR Keywords: “healthy people programs” OR Keywords: “Health Education” OR Keywords: “Community Health Education”)	205
PMC	(Title: (adolescent[Abstract] OR adolescence[Abstract] OR adolescent*[Abstract] OR teen*[Abstract] OR teenager*[Abstract] OR youth*)[Abstract] AND (“Suicidal Ideation”[Abstract] OR “Suicidal Ideations”[Abstract] OR “Self-Injurious Behavior”[Abstract] OR “Deliberate Self Harm”[Abstract] OR “Deliberate Self-Harm”[Abstract] OR “Intentional Self Harm”[Abstract] OR “Intentional Self Injuries”[Abstract] OR “Intentional Self Injury”[Abstract] OR “Non Suicidal Self Injury”[Abstract] OR “Non-Suicidal Self Injuries”[Abstract] OR “Non-Suicidal Self Injury”[Abstract] OR “Nonsuicidal Self Injuries”[Abstract] OR “Nonsuicidal Self Injury”[Abstract] OR “Self Destructive Behavior”[Abstract] OR “Self Harm”[Abstract] OR “Self Injurious Behavior”[Abstract] OR “Self Injury”[Abstract] OR “Self-Destructive Behavior”[Abstract] OR “Self-Destructive Behaviors”[Abstract] OR self-injuries[Abstract] OR “Self-Injurious Behaviors”[Abstract] OR self-injury[Abstract] OR suicide[Abstract] OR suicide*[Abstract] OR “Suicide Prevention”[Abstract] OR “Awareness, Suicide”[Abstract] OR “Prevention, Suicide”[Abstract] OR “Suicide Awareness”[Abstract] OR “Suicide Preventions”)[Abstract] AND (“Health Promotion”[Abstract] OR “Health Campaign”[Abstract] OR “Health Campaigns”[Abstract] OR “Health Promotions”[Abstract] OR “Health-Supportive Environments”[Abstract] OR “Promotion of Health”[Abstract] OR “Promotional Item”[Abstract] OR “Promotional Items”[Abstract] OR “Wellness Program”[Abstract] OR “Wellness Programs”[Abstract] OR “healthy people 2010”[Abstract] OR “healthy people programmes”[Abstract] OR “healthy people programs”[Abstract] OR “Health Education”[Abstract] OR “Community Health Education”)[Abstract])	10
PUBMED	(Adolescent[mh] OR Adolescence OR Adolescent* OR Teen* OR Teenager* OR Youth*) AND (“Suicidal Ideation”[mh] OR “Suicidal Ideations” OR “Self-Injurious Behavior”[mh] OR “Deliberate Self Harm” OR “Deliberate Self-Harm” OR “Intentional Self Harm” OR “Intentional Self Injuries” OR “Intentional Self Injury” OR “Non Suicidal Self Injury” OR “Non-Suicidal Self Injuries” OR “Non-Suicidal Self Injury” OR “Nonsuicidal Self Injuries” OR “Nonsuicidal Self Injury” OR “Self Destructive Behavior” OR “Self Harm” OR “Self Injurious Behavior” OR “Self Injury” OR “Self-Destructive Behavior” OR “Self-Destructive Behaviors” OR Self-Injuries OR “Self-Injurious Behaviors” OR Self-Injury OR Suicide[mh] OR Suicide* OR “Suicide Prevention”[mh] OR “Suicide Awareness” OR “Suicide Preventions”) AND (“Health Promotion”[mh] OR Health Campaign*[tiab] OR Health Promotion*[tiab] OR “Health-Supportive Environments”[tiab] OR “Promotion of Health”[tiab] OR Promotional Item*[tiab] OR Wellness Program*[tiab] OR “healthy people”[tiab] OR healthy people program*[tiab] OR “Health Education”[mh] OR Health Education[tiab])	983
SCIELO	(adolescent OR adolescence OR adolescent* OR teen* OR teenager* OR youth*) AND (“Suicidal Ideation” OR “Suicidal Ideations” OR “Self-Injurious Behavior” OR “Deliberate Self Harm” OR “Deliberate Self-Harm” OR “Intentional Self Harm” OR “Intentional Self Injuries” OR “Intentional Self Injury” OR “Non Suicidal Self Injury” OR “Non-Suicidal Self Injuries” OR “Non-Suicidal Self Injury” OR “Nonsuicidal Self Injuries” OR “Nonsuicidal Self Injury” OR “Self Destructive Behavior” OR “Self Harm” OR “Self Injurious Behavior” OR “Self Injury” OR “Self-Destructive Behavior” OR “Self-Destructive Behaviors” OR self-injuries OR “Self-Injurious Behaviors” OR self-injury OR suicide OR suicide* OR “Suicide Prevention” OR “Awareness, Suicide” OR “Prevention, Suicide” OR “Suicide Awareness” OR “Suicide Preventions”) AND (“Health Promotion” OR “Health Campaign” OR “Health Campaigns” OR “Health Promotions” OR “Health-Supportive Environments” OR “Promotion of Health” OR “Promotional Item” OR “Promotional Items” OR “Wellness Program” OR “Wellness Programs” OR “healthy people 2010” OR “healthy people programmes” OR “healthy people programs” OR “Health Education” OR “Community Health Education”)	6
SCOPUS	(TITLE-ABS-KEY (adolescent OR adolescence OR adolescent* OR teen* OR teenager* OR youth*) AND TITLE-ABS-KEY (“Suicidal Ideation” OR “Suicidal Ideations” OR “Self-Injurious Behavior” OR “Deliberate Self Harm” OR “Deliberate Self-Harm” OR “Intentional Self Harm” OR “Intentional Self Injuries” OR “Intentional Self Injury” OR “Non Suicidal Self Injury” OR “Non-Suicidal Self Injuries” OR “Non-Suicidal Self Injury” OR “Nonsuicidal Self Injuries” OR “Nonsuicidal Self Injury” OR “Self Destructive Behavior” OR “Self Harm” OR “Self Injurious Behavior” OR “Self Injury” OR “Self-Destructive Behavior” OR “Self-Destructive Behaviors” OR self-injuries OR “Self-Injurious Behaviors” OR self-injury OR suicide OR suicide* OR “Suicide Prevention” OR “Awareness, Suicide” OR “Prevention, Suicide” OR “Suicide Awareness” OR “Suicide Preventions”) AND TITLE-ABS-KEY (“Health Promotion” OR “Health Campaign” OR “Health Campaigns” OR “Health Promotions” OR “Health-Supportive Environments” OR “Promotion of Health” OR “Promotional Item” OR “Promotional Items” OR “Wellness Program” OR “Wellness Programs” OR “healthy people 2010” OR “healthy people programmes” OR “healthy people programs” OR “Health Education” OR “Community Health Education”))	1133
WOS	TS=(Adolescent OR Adolescence OR Adolescent* OR Teen* OR Teenager* OR Youth*) AND TS=(“Suicidal Ideation” OR “Suicidal Ideations” OR “Self-Injurious Behavior” OR “Deliberate Self Harm” OR “Deliberate Self-Harm” OR “Intentional Self Harm” OR “Intentional Self Injuries” OR “Intentional Self Injury” OR “Non Suicidal Self Injury” OR “Non-Suicidal Self Injuries” OR “Non-Suicidal Self Injury” OR “Nonsuicidal Self Injuries” OR “Nonsuicidal Self Injury” OR “Self Destructive Behavior” OR “Self Harm” OR “Self Injurious Behavior” OR “Self Injury” OR “Self-Destructive Behavior” OR “Self-Destructive Behaviors” OR Self-Injuries OR “Self-Injurious Behaviors” OR Self-Injury OR Suicide OR Suicide* OR “Suicide Prevention” OR “Awareness, Suicide” OR “Prevention, Suicide” OR “Suicide Awareness” OR “Suicide Preventions”) AND TS=(“Health Promotion” OR “Health Campaign” OR “Health Campaigns” OR “Health Promotions” OR “Health-Supportive Environments” OR “Promotion of Health” OR “Promotional Item” OR “Promotional Items” OR “Wellness Program” OR “Wellness Programs” OR “healthy people 2010” OR “healthy people programmes” OR “healthy people programs” OR “Health Education” OR “Community Health Education”)	338
TOTAL		6583

### Selección de los Estudios

Tras la búsqueda en las bases de datos, las referencias identificadas se importaron a EndNote 21 (Clarivate Analytics, PA, EE. UU.) y se eliminaron los duplicados. Posteriormente, estas referencias se exportaron al *software* Intelligent Systematic Review (Rayyan) para gestionar el proceso de selección. Los documentos se evaluaron por título y resumen según los criterios de inclusión, proceso llevado a cabo por cuatro revisores independientes tras una prueba piloto. Tras la evaluación, se recuperaron y evaluaron en su totalidad las fuentes relevantes, y las decisiones se registraron en Rayyan.

Las discrepancias que surgieron entre los revisores en cada etapa del proceso de selección se resolvieron mediante discusión, consenso y, además, con la intervención de un quinto revisor. Los resultados de la búsqueda y del proceso de inclusión de los estudios se presentan en el diagrama de flujo, de acuerdo con las directrices de PRISMA-ScR^(14)^.

### Extracción y Análisis de Datos

Los datos fueron extraídos de los artículos incluidos por cuatro revisores independientes mediante el uso de una herramienta de extracción de datos (Cuadro 2) desarrollada por los revisores, basada en el manual de la JBI. Los datos incluyeron detalles específicos sobre las características de los estudios (cita, año, país, objetivo, tipo de estudio, muestra), los participantes, el concepto, el contexto (PCC), los métodos de estudio y las principales conclusiones relevantes para la pregunta de la revisión.

**Cuadro 2:** Caracterización de los estudios incluidos en la revisión de alcance – Florianópolis, SC, Brasil, 2025.

Referencia/Año/País		Objetivo	Método/Tipo de estudio	Muestra/Participantes	Entorno de intervención/Contexto	Principales Resultados/Estrategias
1	Klingman y Hochdorf^(18)^, 1993, Israel.	Evaluar la eficacia de un programa escolar de prevención primaria y la formación de los alumnos como guardianes frente a los comportamientos autodestructivos.	Estudio cuasi-experimental con un grupo de intervención y un grupo de control	237 alumnos de 8.º curso (de 12 a 13 años)	Escuela pública	Habilidades para la vida Apoyo entre compañeros
2	Eggert et al.^(19)^, 1994, Estados Unidos.	Evaluar la eficacia de un programa escolar de prevención primaria destinado a reducir el riesgo de suicidio y el consumo de drogas.	Estudio cuasi-experimental con grupo de intervención y grupo de control	Alrededor de 1.000 alumnos de cinco institutos de secundaria	Colegio público	Habilidades para la vida Actividades extraescolares Apoyo entre compañeros
3	LaFromboise y Howard-Pitney^(20)^, 1995, Estados Unidos.	Evaluar la eficacia de un programa de prevención del suicidio centrado en las habilidades para la vida.	Estudio cuasi-experimental con un grupo de intervención y un grupo de control	128 alumnos de secundaria (de 14 a 19 años)	Colegio público	Habilidades para la vida
4	Coggan et al.^(21)^, 1997, Nueva Zelanda.	Investigar las opiniones de los jóvenes sobre el suicidio juvenil	Estudio cualitativo (grupos focales)	140 jóvenes de entre 16 y 24 años	Comunidad, colegios, universidades	Habilidades para la vida Formación para profesores y otros profesionales Campaña educativa Participación y protagonismo Apoyo social y red de apoyo
5	Waring et al.^(22)^, 2000, Australia.	Describir varios proyectos del Hunter Institute of Mental Health, centrados en la promoción y la prevención de la salud mental en adolescentes	Relato de experiencia/Descriptivo.	No hay una muestra específica de participantes, pero incluye la descripción de proyectos dirigidos a estudiantes de secundaria y universitarios	Entornos escolares (bachillerato y universidades), servicios sanitarios, comunidad en general.	Alfabetización en salud mental Apoyo social y red de apoyo Formación para profesores y otros profesionales Cultura y arte Metodología activa/multimodal
6	Day^(23)^, 2004, Reino Unido.	Investigar si la enseñanza de habilidades para la resolución de problemas a adolescentes puede ser una estrategia eficaz en la prevención del suicidio juvenil.	Investigación-acción con un enfoque cualitativo y cuantitativo.	Alumnos de entre 11 y 12 años de 7.º curso, así como profesores, enfermeros escolares y profesionales de la salud mental	Entorno escolar	Habilidades para la vida Coordinación intersectorial
7	Armstrong^(24)^, 2011, Canadá.	Analizar los factores de riesgo y de protección asociados a las ideas suicidas en adolescentes de entornos rurales y urbanos y proponer estrategias de intervención.	Estudio cuantitativo, transversal, descriptivo y analítico.	813 alumnos de secundaria: 459 de zonas rurales y 354 de zonas urbanas	Entorno escolar y comunitario de zonas rurales y urbanas	Participación y protagonismo Actividades extraescolares
8	Carli^(25)^, 2016, Suiza.	Desarrollar y evaluar la eficacia de una intervención *en línea* para la promoción de la salud mental y la prevención del suicidio, dirigida a adolescentes europeos, a través del proyecto SUPREME.	Ensayo clínico aleatorizado, multicéntrico y simple ciego.	2.286 alumnos, de entre 14 y 18 años, de seis países europeos: Suecia, Italia, Inglaterra, España, Lituania, Estonia y Hungría.	Entorno escolar y *en línea* (plataforma virtual desarrollada específicamente para el proyecto SUPREME)	Plataforma digital
9	Silverstone et al.^(26)^, 2017, Canadá.	Evaluar los resultados a largo plazo de un programa escolar multimodal para reducir las ideas suicidas, la depresión y la ansiedad entre los adolescentes.	Estudio cuantitativo, longitudinal y cuasi-experimental.	Inicialmente, 6.227 alumnos Edad: de 11 a 18 años (de 6.º a 12.º curso). 1.884 completaron las cuatro evaluaciones	Colegios públicos	Habilidades para la vida Metodología activa/multimodal Coordinación intersectorial
10	Kahn et al.^(27)^, 2020, Alemania.	Comprender cómo el programa «Youth Aware of Mental Health», una intervención escolar universal para la prevención del suicidio, interactúa con diferentes estrategias de afrontamiento	Ensayo clínico aleatorizado prospectivo	5.654 alumnos, de entre 14 y 16 años, de 85 centros educativos de diez países europeos.	Centros educativos de Europa	Actividades extracurriculares Habilidades y competencias socioemocionales
11	Schilling et al.^(28)^, 2021, Chile.	Determinar cómo las experiencias vitales de los adolescentes contribuyeron a su actuación como expertos por experiencia y al desarrollo del Proyecto Clã, con el objetivo de promover la salud mental y prevenir el suicidio	Estudio de caso cualitativo	Seis alumnos, de entre 13 y 20 años	Centros educativos y entornos digitales en Chile	Apoyo entre iguales Plataforma digital Formación de líderes
12	McGillivray et al.^(29)^, 2021, Australia.	Evaluar el impacto del programa universal «Youth Aware of Mental Health» para la promoción de la salud mental y la prevención del suicidio	Estudio de un solo grupo (*single-arm study*) con pruebas previas y posteriores, de diseño cuasi-experimental	556 alumnos de 3.º de ESO (edades comprendidas entre los 13 y los 16 años), de 18 centros educativos de cuatro regiones de Nueva Gales del Sur, Australia	Centros de enseñanza secundaria.	Habilidades para la vida Metodología activa/multimodal
13	Kaur^(30)^, 2021, India.	Analizar el efecto de la formación en habilidades para la vida sobre el comportamiento suicida de los adolescentes.	Casi-experimental (con grupo de control y grupo de intervención – prueba previa y posterior)	970 alumnos de 3.º de ESO, de 11 centros	Centros públicos de secundaria	Habilidades para la vida Red de apoyo
14	Chou et al.^(31)^, 2022, Canadá.	Evaluar la eficacia del programa Core Connectors Initiative	Estudio de métodos mixtos	29 alumnos, de entre 15 y 17 años	Un colegio público, un colegio privado y un centro comunitario	Alfabetización en salud mental Apoyo entre iguales Red de apoyo
15	Chartier et al.^(32)^, 2022, Canadá.	Analizar, desde la perspectiva de los jóvenes, los mentores y los ancianos de las comunidades indígenas, el enfoque PAX Dream Makers, orientado a la promoción de la salud mental y la prevención del suicidio entre los jóvenes.	Estudio cualitativo, tipo estudio de caso	30 adolescentes (de 12 a 18 años) 17 mentores y ancianos adultos de seis comunidades indígenas remotas	Comunidades indígenas de las Primeras Naciones de Manitoba, en el contexto escolar y comunitario	Habilidades para la vida Plataforma digital Formación de líderes
16	Libonet al.^(33)^, 2023, Canadá.	Recopilar y documentar las perspectivas de los jóvenes sobre el diseño y el desarrollo de un modelo de apoyo entre iguales para la promoción de la salud mental y la prevención del suicidio	Estudio cualitativo descriptivo, con un enfoque de *codiseño* participativo, basado en la estructura socioecológica	11 jóvenes, con edades comprendidas entre los 15 y los 24 años	Comunidades rurales del oeste de Canadá	Apoyo entre iguales Plataforma digital
17	Walsh et al.^(34)^, 2023, Irlanda.	Explorar las perspectivas de los jóvenes sobre la promoción de la salud mental en el ámbito escolar y la prevención del suicidio, utilizando un enfoque participativo (Photovoice)	Estudio cualitativo y participativo, con análisis temático reflexivo y experiencial, basado en el enfoque crítico	15 adolescentes de entre 16 y 19 años	Entorno escolar y comunitario en Irlanda	Apoyo entre iguales
18	Biber y Brandenburg^(35)^, 2024, Estados Unidos.	Describir el proceso de implementación del programa Sources of Strength en un instituto de secundaria, con el objetivo de reforzar los factores de protección de la salud mental y prevenir el comportamiento suicida y el consumo de opioides.	Estudio de caso con evaluación del proceso.	1 250 alumnos de secundaria. 135 alumnos de secundaria actuaron como líderes entre iguales (el 10,8 % del centro). 15 mentores universitarios, 13 profesores y personal del instituto como mentores adultos	Instituto de secundaria	Coordinación intersectorial Formación para profesores y otros profesionales
19	Bohr et al.^(36)^, 2024. Canadá.	Adaptación cultural y evaluación del programa digital SPARX para jóvenes inuit, con el objetivo de fortalecer la salud mental, la resiliencia y la reducción del riesgo de suicidio.	Investigación-acción participativa, con métodos mixtos.	121 jóvenes, de entre 13 y 24 años. 27 líderes juveniles e es (de 15 a 26 años)	Entorno escolar y comunitario, incluidos los espacios virtuales e s.	Habilidades para la vida Cultura y arte Plataforma digital
20	Knappe et al.^(37)^, 2024, Alemania.	Evaluación de la eficacia del programa escolar de prevención del suicidio HEYLiFE	Estudio cuasi-experimental, comparativo, intragrupal (antes y después de la intervención)	218 alumnos, de entre 14 y 18 años	Colegios públicos	Alfabetización en salud mental Metodología activa/multimodal Habilidades para la vida
21	Perrino et al.^(38)^, 2024, Estados Unidos.	Adaptación de una intervención digital para promover la salud mental de los adolescentes hispanos, centrada en la reducción de los síntomas de depresión, ansiedad y riesgo de suicidio.	Estudio de desarrollo y adaptación de una intervención.	16 adolescentes (de 12 a 17 años) y 16 padres.	Centros de atención primaria.	Habilidades para la vida Plataforma digital Participación de los familiares
22	Wexler et al.^(39)^, 2025, Estados Unidos.	Analizar cómo la autoeficacia en el bienestar, la autoeficacia en la prevención del suicidio y la participación en comunidades de práctica influyen en los comportamientos de prevención del suicidio y en la promoción del bienestar.	Estudio transversal, cuantitativo	398 participantes (de entre 15 y 29 años)	Comunidades indígenas rurales y remotas, en espacios comunitarios y redes sociales locales.	Coordinación intersectorial Participación de líderes comunitarios y familiares

Los datos que respondían al objetivo de la revisión se analizaron y presentaron mediante un resumen narrativo, que describía los resultados relacionados con la pregunta de investigación; cabe destacar que se presentaron en forma de cuadro basado en los datos categorizados para facilitar la comprensión.

### Aspectos Éticos

Esta revisión de alcance se ajustó a las directrices metodológicas y éticas aplicables, respetando las buenas prácticas en investigación científica y los derechos de autor de los estudios incluidos. Al tratarse de un análisis de datos secundarios, disponibles públicamente y sin participación de seres humanos ni información identificable, no fue necesario someterlo al Comité de Ética en Investigación, ni obtener un Consentimiento Libre e Informado.

## RESULTADOS

A partir de la aplicación de las estrategias de búsqueda en las bases de datos y portales seleccionados, se obtuvieron un total de 3 884 registros para la lectura de los títulos y resúmenes, de los cuales se seleccionaron 235 estudios para su lectura completa, de acuerdo con los criterios de elegibilidad y la pregunta de investigación. De estos, se seleccionaron 22 estudios para su análisis y discusión, tal como se presenta en el diagrama de flujo PRISMA-ScR de la Figura 1.

**Figura 1:** Diagrama de flujo PRISMA-ScR del proceso de selección de los estudios incluidos – Florianópolis, SC, Brasil, 2025.

Los estudios se publicaron a partir de 1993, con mayor presencia en 2024 (n = 4; 18 %), seguido del año 2021 (n = 3; 14 %). En cuanto a la nacionalidad, destaca Canadá (n = 6; 27 %); le siguen los Estados Unidos (n = 5; 23 %). En cuanto al tipo de estudio, siete (31,5 %) consistieron en estudios cuasi-experimentales; le siguieron los estudios de caso (n = 3; 14 %) y los estudios cualitativos participativos (n = 3; 14 %). En cuanto al escenario, la escuela ocupa un lugar destacado entre los estudios incluidos (n = 18, 82 %). Algunos estudios contaron con más de un escenario, como comunidades rurales, urbanas e indígenas, entornos virtuales y universidades, entre otros, tal como se muestra en el Cuadro 2.

En cuanto a la muestra y los participantes, se observó que la mayoría de los estudios incluidos (n = 18; 82 %) se realizaron con alumnos de escuelas públicas. Sin embargo, algunos también incluyeron a adolescentes procedentes de comunidades rurales e indígenas, así como a maestros, líderes comunitarios, jóvenes adultos y padres. La composición de la muestra varió según el tipo de estudio y sus objetivos, lo que contribuyó a la diversidad de las estrategias de promoción de la salud.

En cuanto a las estrategias de promoción de la salud, surgieron dos enfoques estratégicos, tal como se ilustra en la Figura 2, elaborada mediante la herramienta Google Drawings. Las acciones identificadas se proponen de manera combinada y articulada, con el objetivo de ampliar el alcance y potenciar los efectos de las intervenciones dirigidas a adolescentes con comportamiento suicida. El primer enfoque está dirigido a los adolescentes y destaca estrategias como el desarrollo de habilidades para la vida, el apoyo entre pares, el fortalecimiento de las competencias socioemocionales, la alfabetización en salud mental, el fomento de la participación y el protagonismo juvenil, las actividades extracurriculares y las prácticas artísticas. El segundo enfoque se centra en los diferentes actores sociales involucrados en la atención, lo que incluye campañas educativas, capacitación para maestros y profesionales de diversas áreas, formación de líderes, participación de líderes comunitarios y familiares, fortalecimiento del apoyo social y de las redes de apoyo, coordinación intersectorial, uso de metodologías activas/multimodales y de plataformas digitales.

**Figura 2:** Estrategias de promoción de la salud dirigidas a adolescentes con comportamiento suicida – Florianópolis, SC, Brasil, 2025.

## DISCUSIÓN

Los estudios incluidos en esta revisión de alcance presentaron estrategias de promoción de la salud para adolescentes con comportamiento suicida, con especial énfasis en el entorno escolar, ya que se trata de un contexto propicio tanto para la implementación de estas estrategias como para la prevención del suicidio^(7,40)^. En la escuela, es posible detectar alteraciones, como problemas de conducta, disminución del rendimiento académico y ausentismo frecuente, que pueden ser señales tempranas de sufrimiento psíquico en los adolescentes^(10)^.

Además del entorno escolar, el ámbito comunitario —que incluye contextos urbanos, rurales e indígenas— ha adquirido una importancia similar en la implementación de estrategias de promoción de la salud. En estas comunidades, sobre todo cuando se unen, el fortalecimiento de las redes sociales y la participación en actividades colectivas, como prácticas religiosas y ejercicios en grupo, han demostrado tener el potencial de mejorar el bienestar físico y emocional y de reducir el riesgo de suicidio entre los adolescentes^(39,41)^.

Al mismo tiempo, las plataformas digitales y las redes sociales se perfilan como espacios relevantes para conectar a los adolescentes con sus pares y ofrecerles apoyo emocional^(42)^. Estas herramientas no solo permiten la expresión de sentimientos y experiencias, sino que también favorecen las intervenciones *en línea*, ya que promueven cambios de actitud e incentivan la búsqueda de ayuda^(43,44)^.

Los servicios de atención primaria se han destacado en los entornos escolares, comunitarios y digitales, y se han convertido en escenarios estratégicos para la promoción de la salud mental de los adolescentes con comportamiento suicida, al ampliar el acceso y permitir la implementación de cuidados^(42)^. Aunque aún existen desafíos, se están desarrollando iniciativas como modelos colaborativos y soluciones innovadoras^(45)^. Sin embargo, el estudio señala limitaciones relacionadas con la formación y la experiencia de los profesionales de la atención primaria en el manejo de situaciones como los intentos de suicidio, lo que refuerza la necesidad de capacitación continua y fortalecimiento de los equipos^(46)^.

El mapeo de los escenarios de intervención evidenció que las escuelas, las comunidades, los entornos digitales y la atención primaria amplían el acceso y favorecen la implementación de estrategias de promoción de la salud. Estas estrategias muestran diversidad y abarcan tanto acciones dirigidas a los adolescentes como iniciativas que involucran la participación de otros actores sociales.

En primer lugar, se destaca el desarrollo de habilidades para la vida y competencias socioemocionales, que fortalecen la autonomía, la toma de decisiones, el manejo de las emociones, la resiliencia y la construcción de relaciones interpersonales saludables —elementos fundamentales para la promoción de la salud mental y para la reducción del riesgo de suicidio entre los adolescentes^(18-21,23,29,30,32,36,38)^. Para potenciar estos resultados, estas habilidades deben trabajarse de manera integrada en los contextos escolar, familiar y de los servicios de salud, con énfasis en el uso de los principios de la terapia cognitivo-conductual (TCC), reconocida por su eficacia en el manejo de situaciones relacionadas con el comportamiento suicida y en la promoción de factores de protección^(10,26,36,43,47,48)^.

Otras estrategias implican el fortalecimiento de los grupos de apoyo entre pares, junto con la participación y el protagonismo juvenil, los cuales favorecen el desarrollo psicosocial de los adolescentes al permitir el intercambio de experiencias difíciles y similares. Además, estas iniciativas contribuyen al mejoramiento de las habilidades sociales y relacionales y promueven el empoderamiento juvenil^(28,34,49)^.

Además de estas, otras estrategias resultan relevantes, como la alfabetización en salud mental, las actividades extracurriculares y el uso de la cultura y el arte, que actúan como herramientas poderosas para el fortalecimiento emocional y comunitario de los adolescentes.

La alfabetización en salud mental constituye una base esencial para la promoción y contribuye al bienestar general de los adolescentes. Las intervenciones en esta área han demostrado mejorar habilidades como la autoeficacia y la capacidad de buscar y comprender información sobre la salud, fortaleciendo incluso las conexiones sociales^(7,37)^. También se han aplicado estrategias creativas, como el uso del teatro, para facilitar la alfabetización en salud mental de manera participativa y contextualizada^(22)^. Otro punto significativo es que la cultura y el arte se presentan como estrategias prometedoras y contribuyen positivamente al bienestar al favorecer la expresión de las emociones, la valorización cultural y el fortalecimiento del orgullo identitario, principalmente en comunidades indígenas^(36)^. Las actividades extracurriculares bien estructuradas, como los deportes y la música, también se destacan como moderadoras del estado de ánimo y promotoras de vínculos sociales, ya que refuerzan los factores protectores contra el sufrimiento psíquico^(24)^.

Los estudios se centran directamente en los adolescentes, al tiempo que evidencian la importancia de estrategias que amplíen el alcance de las acciones, como campañas educativas, programas de capacitación para docentes y profesionales de la salud, y la formación de líderes comunitarios y escolares, fundamentales para sostener prácticas de promoción de la salud de manera integrada y continua.

Las campañas de prevención y concientización en los medios de comunicación y en espacios públicos, coordinadas a nivel gubernamental, son esenciales para la prevención y la promoción, y tienen el potencial de influir en las políticas y la legislación en las distintas esferas de gobierno^(47)^. Dirigidas a las comunidades rurales, estas acciones, junto con políticas que amplíen el acceso a actividades estructuradas, fortalecen la participación de los jóvenes en la promoción de la salud^(21,24)^. Como estrategias universales, llegan a un público amplio y ofrecen información esencial para alentar la búsqueda de ayuda^(21)^.

La capacitación, el entrenamiento y la formación deben dirigirse estratégicamente a todos los profesionales y actores sociales, tanto dentro como fuera del sector de la salud, con el objetivo de mejorar los conocimientos y las competencias relacionadas con la salud mental^(47,50)^. Estas estrategias tienen como objetivo, sobre todo, aumentar la concientización, la autoconfianza y la capacidad de los participantes para identificar riesgos de suicidio, intervenir de manera temprana y ofrecer apoyo adecuado^(22,35,44)^. Las evaluaciones de los programas de capacitación ya implementados demuestran su eficacia para fortalecer las prácticas de atención y ampliar la respuesta comunitaria ante situaciones de crisis^(31,43)^.

Además de las campañas, cabe destacar la importancia de la capacitación y la participación de los líderes comunitarios y familiares, con el objetivo de fortalecer la confianza de los adolescentes en sus cuidadores y reparar las rupturas en los vínculos afectivos, factores asociados al riesgo de ideas suicidas y a los intentos de suicidio^(43,44)^. Las estrategias que estimulan la participación familiar y comunitaria, así como el diálogo abierto sobre el suicidio, contribuyen a la creación de entornos sociales acogedores y favorables a la promoción de la salud mental^(7)^. Estos espacios, al permitir la detección temprana de señales de sufrimiento, se vuelven esenciales para la prevención.

La articulación interdisciplinaria e intersectorial entre salud, educación, asistencia social y organizaciones comunitarias potencia las acciones de promoción de la salud y debe fomentarse. Esta integración amplía la participación colectiva en la planificación y el desarrollo de estrategias, de modo que se fortalecen las redes de apoyo y la efectividad de las intervenciones^(23,47)^. El enfoque intersectorial, territorial e interdisciplinario contribuye al fortalecimiento de los factores de protección y, en consecuencia, a la reducción de las tasas de suicidio entre los adolescentes, especialmente cuando existe una colaboración activa entre los sectores y la comunidad^(26,39,51)^.

Las metodologías activas, como los estudios de caso, las dramatizaciones y el aprendizaje basado en problemas, han demostrado resultados prometedores en la capacitación de los adolescentes para el debate y la reflexión sobre el suicidio, pero aún requieren una mayor implementación e integración en los planes de estudio de las materias escolares^(10,22,29,37)^. De manera complementaria, estrategias innovadoras como las plataformas digitales, las redes sociales y las aplicaciones dirigidas a adolescentes y familiares se han destacado por facilitar el aprendizaje de habilidades, ampliar las redes de apoyo y ofrecer servicios *en línea* las 24 horas, lo que las convierte en recursos prometedores para la promoción de la salud mental^(25,28,33,38)^. Sin embargo, estas iniciativas enfrentan desafíos, como cuestiones de accesibilidad, conectividad, seguridad de los datos, necesidad de inversión financiera y adaptaciones culturales para garantizar su efectividad en diferentes contextos^(36,52)^.

Aunque existen marcos normativos sólidos para la implementación de políticas y estrategias de promoción de la salud en Brasil, la articulación institucional, la disponibilidad de recursos, la capacidad técnica y la coordinación intersectorial siguen representando desafíos significativos para su puesta en práctica en el marco del Sistema Único de Salud (SUS)^(53)^. De manera similar, un estudio sobre la promoción de la salud mental indica que la implementación de estrategias eficaces se topa con limitaciones de recursos, la escasez de evidencia sobre la relación costo-efectividad y barreras contextuales que dificultan la adaptación de modelos internacionales a las realidades locales^(54)^.

Las estrategias identificadas en esta revisión refuerzan el papel de la enfermería en la promoción de la salud mental de los adolescentes con comportamiento suicida. Al actuar en diferentes entornos, el personal de enfermería contribuye a ampliar el acceso a la atención, a fortalecer los vínculos y a articular redes de apoyo. Estas acciones exigen una escucha sensible, formación continua y una actuación intersectorial, lo que reafirma el compromiso ético y político de la profesión con la vida y el bienestar de las personas que sufren.

Sin embargo, para fortalecer y ampliar estas prácticas, es necesario reconocer las lagunas que aún existen en la producción científica sobre el tema. Aunque esta revisión de alcance ha identificado diversas estrategias de promoción de la salud para adolescentes con comportamiento suicida, es importante reconocer las limitaciones. Cabe destacar como limitación relevante la imposibilidad de recuperar parte de los estudios identificados, sobre todo los más antiguos. En total, no se pudo acceder a 38 documentos, en su mayoría de las décadas de 1980 y 1990, debido a la falta de disponibilidad del texto completo en las bases de datos y en los repositorios institucionales. A pesar de haber realizado búsquedas complementarias, no fue posible obtenerlos, lo que puede haber reducido el alcance histórico del mapeo.

Además, el predominio de estudios realizados en países de altos ingresos limita la aplicación directa de las estrategias al contexto brasileño, caracterizado por particularidades epidemiológicas, socioculturales y estructurales. Este escenario plantea desafíos para el desarrollo de políticas y prácticas de promoción de la salud dirigidas a adolescentes con comportamiento suicida y refuerza la necesidad de investigaciones nacionales y regionales que consideren las desigualdades socioeconómicas, los determinantes sociales de la salud y la organización de los sistemas públicos, como el SUS. Las investigaciones futuras pueden contribuir a la adaptación, validación y contextualización de las estrategias internacionales, de modo que sean más sensibles a las realidades locales y culturalmente adecuadas.

## CONCLUSIÓN

Los estudios analizados en esta revisión presentan dos enfoques estratégicos que se combinan y articulan para la prevención y la promoción de la salud.

El primero, relacionado directamente con el adolescente, tiene como objetivo fortalecer los recursos internos y relacionales mediante el desarrollo de habilidades para la vida, el apoyo entre pares, las competencias socioemocionales, la alfabetización en salud mental, el estímulo a la participación y al protagonismo juvenil, además de actividades extracurriculares y prácticas artísticas. Estas estrategias contribuyen al empoderamiento de los adolescentes y al fortalecimiento de su resiliencia frente al sufrimiento psíquico.

El segundo enfoque se dirige a diferentes actores sociales que conforman el contexto ampliado de atención, como maestros, profesionales de la salud y la educación, líderes comunitarios y la familia, quienes potencian la creación de entornos de atención y la movilización comunitaria, de manera que se amplíe el alcance y la eficacia de las acciones de promoción de la salud, mediante campañas educativas, capacitación para docentes y profesionales de diversas áreas, formación de líderes, apoyo social y redes de apoyo, articulación intersectorial, uso de metodologías activas/multimodales y plataformas digitales.

Cabe agregar que, si bien el entorno escolar se ha explorado ampliamente como espacio de intervención, se destaca la necesidad de ampliar las estrategias de promoción de la salud a otros contextos, como los servicios especializados en salud mental, a los que muchos adolescentes llegan en una situación crítica de sufrimiento psíquico y con riesgo de suicidio inminente.

También se destaca la necesidad de estrategias que vayan más allá de la capacitación de los profesionales de la salud mediante la incorporación activa de las familias en el proceso de atención.

A pesar del reconocimiento de su papel esencial, se observa una falta de acciones sistemáticas que las incluyan como protagonistas en la promoción de la salud de los adolescentes con comportamiento suicida. Esta brecha pone de manifiesto la urgencia de desarrollar enfoques integrados, intersectoriales y culturalmente sensibles, capaces de impulsar respuestas a las complejas demandas de este público y de contribuir a la reducción de las situaciones de suicidio entre los adolescentes.

## DISPONIBILIDAD DE DATOS

Los datos que respaldan los resultados de este estudio están disponibles de forma abierta en el Open Science Framework (OSF), en: https://doi.org/10.17605/OSF.IO/J9MQB.
